# Synthesis and Evaluation of the Acetylcholinesterase Inhibitory Activities of Some Flavonoids Derived from Naringenin

**DOI:** 10.1155/2021/4817900

**Published:** 2021-11-30

**Authors:** The-Huan Tran, Thi-Thu-Hien Vo, Thi-Quynh-Nhi Vo, Thi-Cam-Nhung Cao, Thai-Son Tran

**Affiliations:** Department of Pharmaceutical Chemistry, Faculty of Pharmacy, University of Medicine and Pharmacy, Hue University, Hue 530000, Vietnam

## Abstract

Alzheimer's disease (AD) is an irreversible neurodegenerative disease that affects many older people adversely. AD has been putting a huge socioeconomic burden on the healthcare systems of many developed countries with aging populations. The need for new therapies that can halt or reverse the progression of the disease is now extremely great. A research approach in the finding new treatment for AD that has attracted much interest from scientists for a long time is the reestablishment of cholinergic transmission through inhibition of acetylcholinesterase (AChE). Naringenin is a flavonoid with the potential inhibitory activity against AChE. From naringenin, many other flavonoid derivatives, such as flavanones and chalcones, can be synthesized. In this study, by applying the Williamson method, nine flavonoid derivatives were synthesized, including four flavanones and five chalcones. The evaluation of AChE inhibitory activity by the Ellman method showed that there were four substances (**2, 4, 5,** and **7**) with relatively good biological activities (IC_50_ < 100 *μ*M), and these biological activities were better than that of naringenin. The molecular docking revealed that strong interactions with amino acid residue Ser200 of the catalytic triad and those of the peripheral region of the enzyme were crucial for strong effects against AChE. Compound **7** had the strongest AChE inhibitory activity (IC_50_ 13.0 ± 1.9 *μ*M). This substance could be used for further studies.

## 1. Introduction

Alzheimer's disease (AD) is one of the greatest medical challenges facing us in the current era. This is the most common form of dementia. The disease is manifested by intellectual impairment, memory loss, language disorders, and the disability in movements as well as cognition, leading to serious impacts on occupational activities and social communication of the patients. AD also has a heavy impact on the patient's family and people around because caring for an AD patient is often difficult and expensive. AD worsens over time and eventually leads to death [1]. Worldwide, the number of people living with AD-related dementia is around 50 million (2018), and this figure is expected to grow to 152 million by 2050 [2], with trillions of US dollars paid for the global healthcare for the disease [3].

The cholinergic hypothesis suggests that cholinergic neurotransmission is likely to play a vital role in memory, learning, concentration, and other advanced neural functions. Several studies have suggested additional roles of the cholinergic system in overall brain homeostasis and neural plasticity. Thus, the cholinergic system has occupied a central place in many research studies related to normal cognitive function and age-related dementia, including AD [4]. Acetylcholinesterase (AChE, EC 3.1.1.7) catalyzes the hydrolysis of acetylcholine and some other neurotransmitter choline esters. AChE is found primarily at cholinergic synapses, where it rapidly degrades acetylcholine to release choline and acetate. Hence, AChE plays an essential role in cholinergic neurotransmission. Acetylcholinesterase inhibitors increase the levels of acetylcholine in synaptic clefts and are one of the very few proven clinically effective therapies in the treatment of AD-induced dementia. Therefore, AChE is an important therapeutic target for the disease currently [5–8].

Flavonoid is a large group of naturally occurring compounds with diverse biological activities [9]. Naringenin is a natural flavonoid found in many plants [10]. As a flavonoid, this compound has many biological activities such as antioxidant [11], anti-inflammatory [12], antiviral [13], and anticancer [14]. In particular, naringenin has been reported to have AChE inhibitory activity [15].

In this study, flavonoid derivatives were synthesized from naringenin and were evaluated for their inhibitory effects on AChE. The mode of interaction to the enzyme target of these compounds and their predicted profiles in physicochemical properties, pharmacokinetics, and toxicities were also investigated to serve as premises for further studies.

## 2. Experimental

### 2.1. Materials and Methods

All chemicals were obtained from commercial suppliers and used without further purification. Melting points determination, UV (ultraviolet), IR (infrared), HR-MS (high-resolution mass spectrometry), ^1^H-NMR (proton nuclear magnetic resonance), and ^13^C-NMR (carbon-13 nuclear magnetic resonance) spectra elucidation and all computation processes were performed on the systems as described earlier [16].

### 2.2. Synthesis of Flavonoid Derivatives from Naringenin

**Scheme 1:**
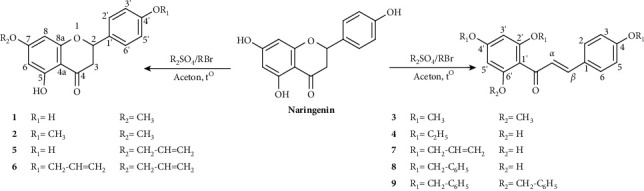
Synthesis of flavonoid derivatives from naringenin.

The Williamson method [17] was applied for the synthesis of flavonoid derivatives from naringenin. The reactions are indicated in Scheme 1 and are described briefly as follows: naringenin was dissolved in acetone. K_2_CO_3_ and dialkyl sulfate or alkyl bromide were then added. The reaction mixture was heated at 40–45°C and was monitored by thin layer chromatography with the appropriate solvent systems. At the end of the reaction, the mixture was filtered to remove the insoluble solid. The obtained solution was evaporated under reduced pressure. The crude product was purified by recrystallization or by column chromatography with the appropriate solvent systems. The structures of the synthetic substances were elucidated by UV, IR, HR-MS, ^1^H-NMR, and ^13^C-NMR spectra.

#### 2.2.1. 4′,5-Dihydroxy-7-methoxyflavanone (**1**)

White, needle-shaped crystals slightly soluble in water, soluble in methanol, highly soluble in acetone, and sparingly soluble in dichloromethane. Yield is 54%. Melting point: 106-107°C. UV (*λ*_max_ nm, MeOH): 287. *IR* (*ν* cm^−1^, KBr): 3523 (*ν*_OH_), 3115 (*ν*_C–H sp2_), 2893 (*ν*_C–H sp3_), 1640 (*ν*_C=O_), 1618 (*ν*_C=C_), 1444 (*ν*_C=C_), and 1153 (*ν*_C–O_). HR-MS (ESI) m/z: [M + H]^+^ calculated for C_16_H_15_O_5_ 287.0919; found 287.0911. ^1^H-NMR (600 MHz, DMSO-*d*_6_): *δ* 12.11 (s, 1H, OH); 9.58 (s, 1H, OH); 7.33–7.31 (m, 2H, H_Ar_); 6.80–6.78 (m, 2H, H_Ar_); 6.10 (d, *J* = 2.4 Hz, 1H, H_Ar_); 6.08 (d, *J* = 2.4 Hz, 1H, H_Ar_); 5.49 (dd, *J* = 12.6; 3.0 Hz, 1H, O–CH<); 3.78 (s, 3H, CH_3_); 3.32 (dd, *J* = 17.4; 12.6 Hz, 1H, CH_2_); and 2.72 (dd, *J* = 17.4; 3.0 Hz, 1H, CH_2_). ^13^C-NMR (150 MHz, DMSO-*d*_6_): *δ* 196.9 (C=O), 167.4, 163.1, 162.8, 157.7, 128.6, 128.3, 115.1, 102.5, 94.6, 93.7, 78.6 (O–CH<), 55.8 (CH_3_), and 42.0 (CH_2_).

#### 2.2.2. 5-Hydroxy-4′,7-dimethoxyflavanone (**2**)

White, needle-shaped crystals insoluble in water, sparingly soluble in methanol, soluble in acetone, and highly soluble in dichloromethane. Yield is 68%. Melting point: 116-117°C. UV (*λ*_max_ nm, MeOH): 288. IR (*ν* cm^−1^, KBr): 3446 (*ν*_OH_), 3093 (*ν*_C–H sp2_), 2914 (*ν*_C–H sp3_), 1631 (*ν*_C=O_), 1579 (*ν*_C=C_), 1433 (*ν*_C=C_), and 1157 (*ν*_C–O_). HR-MS (ESI) m/z: [M + H]^+^ calculated for C_17_H_17_O_5_ 301.1076; found 301.1066. ^1^H-NMR (500 MHz, DMSO-*d*_6_): *δ* 12.10 (s, 1H, OH); 7.46–7.43 (m, 2H, H_Ar_); 6.99–6.96 (m, 2H, H_Ar_); 6.12 (d, *J* = 2.0 Hz, 1H, H_Ar_); 6.08 (d, *J* = 2.0 Hz, 1H, H_Ar_); 5.55 (dd, *J* = 13.0; 3.0 Hz, 1H, O–CH<); 3.79 (s, 3H, CH_3_); 3.77 (s, 3H, CH_3_); 3.33 (dd, *J* = 17.0; 13.0 Hz, 1H, CH_2_); and 2.77 (dd, J = 17.0; 3.0 Hz, 1H, CH_2_). ^13^C-NMR (125 MHz, DMSO-*d*_6_): *δ* 196.7 (C=O), 167.4, 163.1, 162.7, 159.4, 130.4, 128.2, 113.8, 102.5, 94.6, 93.7, 78.3 (O–CH<), 55.8 (CH_3_), 55.1 (CH_3_), and 42.0 (CH_2_).

#### 2.2.3. (E)-2′,4,4′,6′-Tetramethoxychalcone (**3**)

Green, needle-shaped crystals insoluble in water, sparingly soluble in methanol, soluble in acetone, and highly soluble in dichloromethane. Yield is 47%. Melting point: 123-124°C. UV (*λ*_max_ nm, MeOH): 327. IR (*ν* cm^−1^, KBr): 3008 (*ν*_C–H sp2_), 2937 (*ν*_C–H sp3_), 1672 (*ν*_C=O_), 1600 (*ν*_C=C_), 1465 (*ν*_C=C_), and 1126 (*ν*_C–O_). HR-MS (ESI) m/z: [M + H]^+^ calculated for C_19_H_21_O_5_ 329.1389; found 329.1389. ^1^H-NMR (500 MHz, DMSO-*d*_6_): *δ* 7.61–7.59 (m, 2H, H_Ar_); 7.18 (d, *J* = 16.0 Hz, 1H, H_*β*_); 6.95–6.94 (m, 2H, H_Ar_); 6.83 (d, *J* = 16.0 Hz, 1H, H_*α*_); 6.31 (s, 2H, H_Ar_); 3.83 (s, 3H, CH_3_); 3.78 (s, 3H, CH_3_); and 3.71 (s, 6H, CH_3_). ^13^C-NMR (125 MHz, DMSO-*d*_6_): *δ* 193.2 (C=O), 161.7, 161.1, 157.9, 143.6, 130.2, 126.9, 126.8, 114.4, 111.2, 91.0, 55.7 (CH_3_), 55.3 (CH_3_), and 55.2 (CH_3_).

#### 2.2.4. (E)-2′,4,4′-Triethoxy-6′-hydroxychalcone (**4**)

Yellow, needle-shaped crystals insoluble in water, slightly soluble in methanol, soluble in acetone, and highly soluble in dichloromethane. Yield is 52%. Melting point: 135-136°C. UV (*λ*_max_ nm, MeOH): 364. IR (*ν* cm^−1^, KBr): 3446 (*ν*_OH_), 2981 (*ν*_C–H sp2_), 2933 (*ν*_C–H sp3_), 1622 (*ν*_C=O_), 1554 (*ν*_C=C_), 1473 (*ν*_C=C_), and 1118 (*ν*_C–O_). HR-MS (ESI) m/z: [M + H]^+^ calculated for C_21_H_25_O_5_ 357.1702; found 357.1702. ^*1*^*H-NMR* (500 MHz, DMSO-*d*_6_): *δ* 13.71 (s, 1H, OH); 7.78 (d, *J* = 15.5 Hz, 1H, H_*β*_); 7.63 (d, *J* = 15.5 Hz, 1H, H_*α*_); 7.61–7.59 (m, 2H, H_Ar_); 6.99–6.97 (m, 2H, H_Ar_); 6.08 (d, *J* = 2.0 Hz, H_Ar_); 6.06 (d, *J* = 2.0 Hz, H_Ar_); 4.16–4.07 (m, 6H, CH_2_); 1.42 (t, *J* = 7.0 Hz, 3H, CH_3_); 1.34 (t, *J* = 7.0 Hz, 3H, CH_3_); and 1.33 (t, J = 7.0 Hz, 3H, CH_3_). ^13^C-NMR (125 MHz, DMSO-*d*_6_): *δ* 191.8 (C=O), 165.9, 164.6, 161.1, 160.2, 141.5, 129.6, 127.2, 124.8, 114.7, 105.9, 94.1, 91.7, 64.2 (CH_2_), 63.3 (CH_2_), 63.0 (CH_2_), 14.1 (CH_3_), and 13.9 (CH_3_).

#### 2.2.5. 7-Allyloxy-4′,5-dihydroxyflavanone (**5**)

Yellow powder and insoluble in water, sparingly soluble in methanol and in dichloromethane, and highly soluble in acetone. Yield is 58%. Melting point: 103-104°C. *UV* (*λ*_max_ nm, MeOH): 288. IR (*ν* cm^−1^, KBr): 3406 (*ν*_OH_), 3024 (*ν*_C–H sp2_), 2918 (*ν*_C–H sp3_), 1635 (*ν*_C=O_), 1570 (*ν*_C=C_), 1446 (*ν*_C=C_), and 1166 (*ν*_C–O_). HR-MS (ESI) m/z: [M+H]^+^ calculated for C_18_H_17_O_5_ 313.1076; found 313.1076. ^1^H-NMR (600 MHz, DMSO-*d*_6_): *δ* 12.09 (s, 1H, OH); 9.59 (s, 1H, OH); 7.32 (d, *J* = 8.4 Hz, 2H, H_Ar_); 6.79 (d, J = 8.4 Hz, 2H, H_Ar_); 6.11 (d, *J* = 2.4 Hz, 1H, H_Ar_); 6.09 (d, *J* = 2.4 Hz, 1H, H_Ar_); 6.01–5.97 (m, 1H, CH=); 5.48 (dd, *J* =  = 12.6; 3.0 Hz, 1H, O–CH<); 5.37 (dd, *J* =  = 17.4; 1.8 Hz, 1H, CH_2_=); 5.26 (dd, *J* = 10.8; 1.8 Hz, 1H, CH_2_=); 4.62 (d, *J* = 5.4 Hz, 2H, O–CH_2_–); 3.32 (dd, J = 17.4; 12.6 Hz, 1H, –CH_2_–); and 2.71 (dd, *J* = 17.4; 3.0 Hz, 1H, –CH_2_–). ^13^C-NMR (150 MHz, DMSO-*d*_6_): *δ* 196.9 (C=O), 166.2, 163.1, 162.8, 157.7, 132.8, 128.6, 128.3, 118.0, 115.1, 102.6, 95.2, 94.3, 78.6 (O–CH<), 68.7 (O–CH_2_), and 42.0 (CH_2_).

#### 2.2.6. 4′,7-Diallyloxy-5-hydroxyflavanone (**6**)

Yellow powder and insoluble in water, sparingly soluble in methanol, soluble in acetone, and highly soluble in dichloromethane. Yield is 61%. Melting point: 61-62°C. UV (*λ*_max_ nm, MeOH): 288. IR (*ν* cm^−1^, KBr): 3446 (*ν*_OH_), 3080 (*ν*_C–H sp2_), 2916 (*ν*_C–H sp3_), 1651 (*ν*_C=O_), 1616 (*ν*_C=C_), 1514 (*ν*_C=C_), and 1170 (*ν*_C–O_). HR-MS (ESI) m/z: [M + H]^+^ calculated for C_21_H_21_O_5_ 353.1389; found 353.1388. ^1^H-NMR (600 MHz, DMSO-*d*_6_): *δ* 12.08 (s, 1H, OH); 7.44 (d, *J* = 8.4 Hz, 2H, H_Ar_); 7.00 (d, *J* = 8.4 Hz, 2H, H_Ar_); 6.13 (d, *J* = 2.4 Hz, 1H, H_Ar_); 6.10 (d, *J* = 2.4 Hz, 1H, H_Ar_); 6.07–5.96 (m, 2H, CH=); 5.55 (dd, J = 12.6; 3.0 Hz, 1H, O–CH<); 5.41–5.36 (m, 2H, CH_2_=); 5.27–5.25 (m, 2H, CH_2_=); 4.63 (d, *J* = 5.4 Hz, 2H, O–CH_2_–); 4.58 (d, *J* = 5.4 Hz, 2H, O–CH2–); 3.34 (dd, *J* = 17.4; 12.6 Hz, 1H, CH_2_); and 2.76 (dd, *J* = 17.4; 3.0 Hz, 1H, CH_2_). ^13^C-NMR (150 MHz, DMSO-*d*_6_): *δ* 196.7 (C=O), 166.2, 163.1, 162.7, 158.3, 133.5, 132.8, 130.5, 128.2, 118.0, 117.4, 114.6, 102.6, 95.2, 94.3, 78.3 (O–CH<), 68.7 (O–CH_2_), 68.1 (O–CH_2_), and 41.9 (CH_2_).

#### 2.2.7. (E)-2′,4,4′-Triallyloxy-6′-hydroxychalcone (**7**)

Yellow, needle-shaped crystals insoluble in water, sparingly soluble in methanol, soluble in acetone, and highly soluble in dichloromethane. Yield is 66%. Melting point: 72-73°C. UV (*λ*_max_ nm, MeOH): 364. IR (*ν* cm^−1^, KBr): 3452 (*ν*_OH_), 3080 (*ν*_C–H sp2_), 2933 (*ν*_C–H sp3_), 1627 (*ν*_C=O_), 1554 (*ν*_C=C_), 1423 (*ν*_C=C_), and 1166 (*ν*_C–O_). HR-MS (ESI) m/z: [M + H]^+^ calculated for C_24_H_25_O_5_ 393.1702; found 393.1707. ^1^H-NMR (500 MHz, DMSO-*d*_6_): *δ* 13.60 (s, 1H, OH); 7.73 (d, *J* = 15.5 Hz, 1H, H_*β*_); 7.64 (d, *J* = 15.5 Hz, 1H, H_*α*_); 7.61 (d, *J* = 9.0 Hz, 2H, H_Ar_); 7.00 (d, *J* = 9.0 Hz, 2H, H_Ar_); 6.12 (d, *J* = 2.5 Hz, 1H, H_Ar_); 6.17 (d, *J* = 2.5 Hz, 1H, H_Ar_); 6.14–5.99 (m, 3H, O–CH=); 5.46–5.38 (m, 3H, =CH_2_); 5.32–5.26 (m, 3H, =CH_2_); 4.66 (d, *J* = 5.5 Hz, 2H, CH_2_); 4.62 (d, *J* = 5.5 Hz, 2H, CH_2_); and 4.61 (d, *J* = 5.5 Hz, 2H, CH_2_). ^13^C-NMR (125 MHz, DMSO-*d*_6_): *δ* 192.0 (C=O), 165.6, 164.2, 160.7, 160.1, 142.2, 133.2, 132.9, 132.8, 130.1, 127.4, 125.0, 118.4, 117.9, 117.6, 115.1, 106.4, 94.7, 92.4, 69.3 (CH_2_), 68.5 (CH_2_), and 68.2 (CH_2_).

#### 2.2.8. (E)-2′,4,4′-Tribenzyloxy-6′-hydroxychalcone (**8**)

Yellow powder and insoluble in water and in methanol and soluble in acetone and in dichloromethane. Yield is 57%. Melting point*:* 140-141°C. UV (*λ*_max_ nm, MeOH): 370. IR (*ν* cm^−1^, KBr): 3446 (*ν*_OH_), 3037 (*ν*_C–H sp2_), 2943 (*ν*_C–H sp3_), 2881 (*ν*_C–H sp3_), 1622 (*ν*_C=O_), 1562 (*ν*_C=C_), 1421 (*ν*_C=C_), and 1157 (*ν*_C–O_). HR-MS (ESI) m/z: [M + H]^+^ calculated for C_36_H_31_O_5_ 543.2171; found 543.2168. ^1^H-NMR (600 MHz, DMSO-*d*_6_): *δ* 14.11 (s, 1H, OH); 7.68 (d, *J* = 15.6 Hz, 1H, H_*β*_); 7.60 (d, *J* = 15.6 Hz, 1H, H_*α*_); 7.56–7.54 (m, 2H, H_Ar_); 7.48–7.46 (m, 4H, HAr); 7.43–7.35 (m, 9H, H_Ar_); 7.14 (d, *J* = 8.4 Hz, 2H, H_Ar_); 6.90 (d, *J* = 8.4 Hz, 2H, H_Ar_); 6.40 (d, *J* = 2.4 Hz, 1H, H_Ar_); 6.24 (d, *J* = 2.4 Hz, 1H, H_Ar_); 5.21 (s, 2H, CH_2_); 5.17 (s, 2H, CH_2_); and 5.16 (s, 2H, CH_2_). ^13^C-NMR (150 MHz, DMSO-*d*_6_): *δ* 191.9 (C=O), 166.7, 164.7, 161.2, 160.1, 142.7, 136.6, 136.2, 135.7, 130.1, 128.9, 128.7, 128.5, 128.4, 128.1, 127.9, 127.8, 127.7, 127.3, 124.6, 115.1, 105.9, 94.9, 92.6, 71.0 (CH_2_), 69.7 (CH_2_), and 69.3 (CH_2_).

#### 2.2.9. (E)-2′,4,4′,6′-Tetrabenzyloxychalcone (**9**)

White, needle-shaped crystal insoluble in water, slightly soluble in methanol, soluble in acetone, highly soluble in dichloromethane. Yield is 67%. Melting point: 139-140°C. UV (*λ*_max_ nm, MeOH): 330. IR (*ν* cm^−1^, KBr): 3446 (*ν*_OH_), 3028 (*ν*_C–H sp2_), 2933 (*ν*_C–H sp3_), 2864 (*ν*_C–H sp3_), 1633 (*ν*_C=O_), 1597 (*ν*_C=C_), 1431 (*ν*_C=C_), and 1124 (*ν*_C–O_). HR-MS (ESI) m/z: [M+H]^+^ calculated for C_43_H_37_O_5_ 633.2641; found 633.2633. ^1^H-NMR (600 MHz, DMSO-d_6_): *δ* 7.61 (d, *J* = 8.4 Hz, 2H, H_Ar_); 7.46–7.45 (m, 4H, H_Ar_); 7.42–7.38 (m, 4H, H_Ar_); 7.37–7.33 (m, 2H, H_Ar_); 7.31–7.29 (m, 4H, H_Ar_); 7.24–7.20 (m, 6H, H_Ar_); 7.22 (d, *J* = 16.2 Hz, 1H, H_*β*_), 7.05 (d, *J* = 8.4 Hz, 2H, H_Ar_); 6.92 (d, *J* = 16.2 Hz, 1H, H_*α*_); 6.51 (s, 2H, H_Ar_); 5.17 (s, 2H, CH_2_); 5.13 (s, 2H, CH_2_); and 5.10 (s, 4H, CH_2_). ^13^C-NMR (150 MHz, DMSO-*d*_6_): *δ* 192.9 (C=O), 160.6, 160.2, 156.9, 143.6, 136.8, 136.6, 136.6, 131.6, 130.2, 128.4, 128.4, 128.3, 128.2, 128.0, 127.9, 127.9, 127.8, 127.7, 127.6, 127.2, 127.1, 127.1, 126.9, 115.2, 112.4, 93.4, 69.7 (CH_2_), 69.6 (CH_2_), 69.5 (CH_2_), and 69.3 (CH_2_).

All the spectral data of the synthesized compounds are provided in the Supplementary Materials (Tables S1–S9).

### 2.3. Acetylcholinesterase Inhibition Assay

AChE inhibitory activity was determined using the materials and methods as described earlier [16]. The initial mixture in each well consisted of phosphate buffer pH 8, sample (studied compounds or reference) prepared at different concentrations in dimethyl sulfoxide, and AChE enzyme solution 0.25 UI/mL (in phosphate buffer). This mixture was incubated at 25°C for 15 minutes, and the solutions of 2.4 mM 5.5-dithio-bis-2-nitrobenzoic acid (reagent solution) and 2.4 mM acetylthiocholine iodide (substrate solution) were then added. Continue incubating the mixture at 25°C for 24 minutes, and then, the absorbance was measured at 405 nm. All samples were assayed in triplicate.

### 2.4. Molecular Docking Study

The methods and software are used in molecular docking study as described in the previous work [16]. Computational programs including Sybyl-X 2.0 [18], FlexX [19], and MOE 2008.10 [20] were used with default settings for ligands preparation, docking procedure, and interactions analysis, respectively. Protein complex 1W6R of AChE (acetylcholinesterase from *Tetronarce californica* in complex with the bound ligand, (-)-galantamine, at a resolution of 2.05 Å) was downloaded from Protein Data Bank [21] and utilized in this study. Docking protocols were validated by the method of pose selection [22], and the root mean square deviation (RMSD) value between redocked conformations and the original bound ligand in the cocrystallized complex which was ≤1.5 Å would confirm the reliability of the binding ability prediction of new ligands.

### 2.5. Prediction of Physicochemical Properties, Pharmacokinetics, and Toxicities

Physicochemical properties, pharmacokinetics, drug-likeness, and medicinal chemistry features of synthesized compounds and galantamine were predicted using the free online service SwissADME (http://www.swissadme.ch/) [23]. Predicted toxicities of studied substances were obtained using ProTox-II web server (https://tox-new.charite.de/protox_II/) [24].

The parameters calculated by the two mentioned computational tools are given in Tables 1 and 2.

## 3. Results and Discussion

### 3.1. Synthesis of Naringenin Derivatives

From naringenin, the reactions of etherification (with acetone as solvent and K_2_CO_3_ as the catalyst) were conducted (Scheme 1) to produce nine flavonoid derivatives with relatively high yields (47–68%). Two out of these compounds (**6** and **9**) were found as completely new structures (according to search results on SciFinder on March 8, 2021).

Naringenin has acidic phenolic OH groups which in alkaline condition (like K_2_CO_3_) are easily converted into anionic phenolate (Ar–O^−^) with higher electron density than OH groups, as increased ability to participate in electrophilic substitution reactions. Anhydrous acetone is a good solvent that dissolves raw materials and formed products and limits the decomposition of unstable agents such as dimethyl sulfate and diethyl sulfate.

**Scheme 2:**
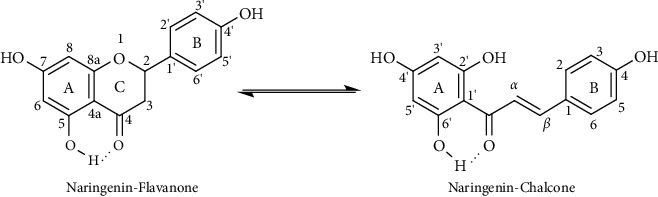
The conversion between flavanone and chalcone.

Flavanones can be easily converted to isomeric chalcones in alkaline (or acidic) media, provided within there is a hydroxyl substituent at position 2′ (or 6′) of chalcones (Scheme 2) [25]. Naringenin is a flavonoid with a chemical scaffold of flavanone, and opening ring C will form a chalcone derivative with OH groups at the 2′ and 6′ positions, respectively. In addition, the OH group at position 5 of the A ring of naringenin has an intramolecular hydrogen bond with C=O at position 4 of the C ring, so this position is more difficult to be substituted than other positions. To etherify the OH group at this position, it is necessary to give more K_2_CO_3_ catalyst and perform the reaction at higher temperatures and for a longer time. With the above reaction conditions, naringenin is easy to open C ring to form chalcone derivatives. This was proven by experiments creating compounds **3**, **4**, **7**, **8,** and **9**.

The structures of synthetic substances were elucidated by UV, IR, HR-MS, ^1^H-NMR, and ^13^C-NMR spectra. The UV spectra all showed characteristic maximum absorption peaks in the wavelength region of 270–295 nm for flavanones (**1, 2, 5,** and **6**), and 330–390 nm for chalcones (**3, 4, 7, 8,** and **9**). The HR-MS spectra of the synthesized compounds were in agreement with the expected data with very small deviations.

All IR spectra have absorption bands at the wavenumbers corresponding to the characteristic functional groups of flavonoids. In which, the vibrations of the bonds O–H, C=O, C=C, C–O, C–H are typical for the studied derivatives. The OH group has a signal at the wavenumber of 3400–3600 cm^−1^. Since the C–H_sp2_ bond is stronger than that in C–H_sp3_, this group (CH_sp2_) stretches at higher frequencies. On the IR spectra, there appears one or more peaks located in the 3000 cm^−1^ region, which is typical for C–H_sp2_ bond. The signals of C–H_sp3_ give peaks in the region near 2900 cm^−1^. The C=O_ketone_ group in ring C is among the easily recognizable functional groups in the IR spectra, and this group undergoes vibrational excitation at the wavenumber of 1600–1700 cm^−1^. A strong absorption band is also observed between 1450 and 1600 cm^−1^ that characterizes the double bonds of the aromatic ring.

Regarding thermodynamic equilibrium between flavanones and chalcones (Scheme 2), the spectral signals with chemical shifts of approximately *δ* 5.49 ppm (dd, *J* =  = 12.6; 3.0 Hz, 1H, O–CH<), *δ* 3.32 ppm (dd, J = 17.4; 12.6 Hz, 1H, CH2), *δ* 2.72 ppm (dd, *J* = 17.4; 3.0 Hz, 1H, CH_2_) in ^1^H-NMR spectra, and *δ* 196.9 ppm (C=O) in ^13^C-NMR spectra indicate that obtained derivatives have a flavanone scaffold (compound **1** and similarly for **2, 5,** and **6**) [26]. The spectral signals with chemical shifts of approximately *δ* 7.78 ppm (d, *J* =15.5 Hz, 1H, H_*β*_) and *δ* 7.63 (d, *J* = 15.5 Hz, 1H, H_*α*_) confirm the chalcone derivatives (compound **4** and similarly for **3, 7, 8,** and **9**) [27]. At the same time, with the *J* value of H *α*&*β* is approximately 16.0 Hz, these chalcone derivatives all have the *E* configuration.

In the ^1^H-NMR spectra of the flavanone derivatives, in addition to the signal peaks typical for this scaffold mentioned above, there are also characteristic peaks for each substance. Specifically, in monoetherified compounds, there are two singlet signals of two OH protons in the low magnetic field region, in which OH at C_5_ forms an intramolecular hydrogen bond with the C=O_ketone_ group of the C ring, reducing the electron density around this proton, so the signal of this proton will be in a lower magnetic field region than the OH proton at C_4′_. Substances with 2 etherified OH groups give a singlet signal in the low magnetic field region, while for fully etherified derivatives, this signal no longer appears on the spectra.

The ^13^C-NMR spectra of the derivatives all give characteristic peaks of C=O_ketone_, aromatic rings, or substituents. Due to the symmetry in the B ring (C_2′_ vs. C_6′_ and C_3′_ vs. C_5′_ in flavanones and C_2_ vs. C_6_ and C_3_ vs. C_5_ in chalcones), there is an overlap signal between these carbon peaks. Therefore, there were four peaks for six aromatic carbons (ring B) on the spectra. Among them, a peak with the smallest intensity is that of carbon C_1'_ (or C_1_) because this carbon has no attached hydrogen. Two stronger signals are for C_2′_ and C_6′_ (or C_2_ and C_6_) and C_3′_ and C_5′_ (or C_3_ and C_5_). The medium intensity peak is for C_4′_ (or C_4_).

### 3.2. In Vitro Assay for Acetylcholinesterase Inhibition

Bioactivity assays were conducted on eleven samples, including nine synthetic derivatives, naringenin, and galantamine as a positive control. The results are given in Table 3.

The results showed that all studied flavonoids had weaker AChE inhibitory activities than that of galantamine. Two new substances (**6** and **9**) had negligible biological effects. Four substances have improved activities over naringenin. These substances are **2, 4, 5,** and **7,** which all have IC_50_ under 100 *μ*M. Compound **7** had the best inhibitory activity against AChE in the synthesized flavonoids with the IC_50_ value of 13.0 ± 1.9 *μ*M. The two most active substances (**4** and **7**) were found to be chalcones derived from flavanones by ring opening and with the OH group at C_5_ position not being etherified.

### 3.3. Molecular Docking

**Figure 1:**
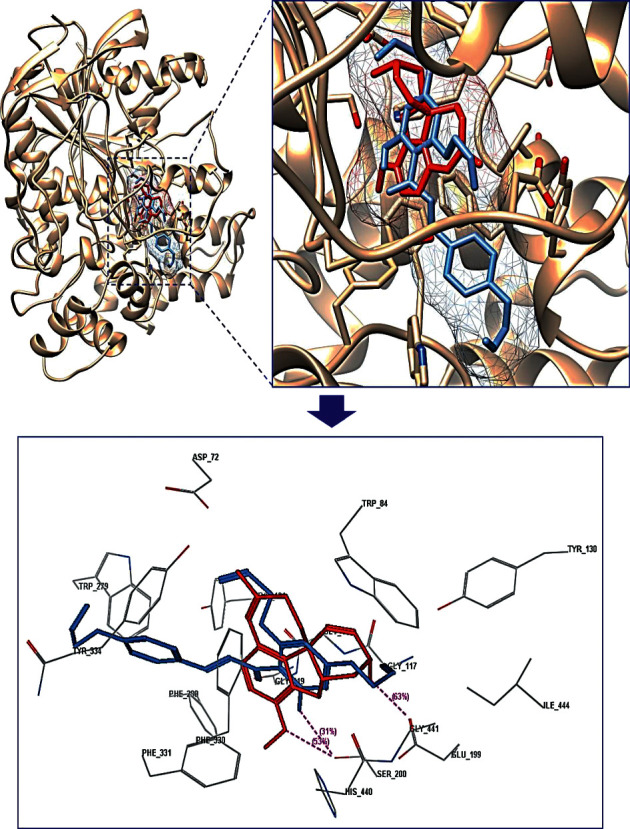
Alignment of docked pose of compound **7** with galantamine as cocrystallized ligand in the AChE complex (PDB ID: 1W6R). Compound **7** is rendered in cornflower blue, and galantamine is rendered in red. Asp, aspartate; Trp, tryptophan; Gly, glycine; Tyr, tyrosine; Glu, glutamate; Ser, serine; Phe, phenylalanine; His, histidine; Ile, isoleucine.

**Figure 2:**
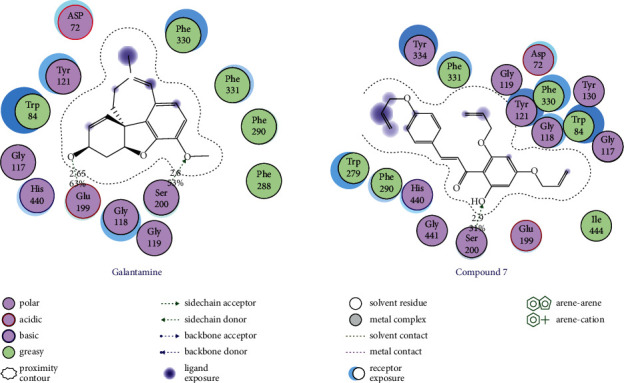
Mode of interaction of galantamine and compound 7 with the enzyme AChE (depicted in two-dimensional format). Asp, aspartate; Trp, tryptophan; Gly, glycine; Tyr, tyrosine; Glu, glutamate; Ser, serine; Phe, phenylalanine; His, histidine; Ile, isoleucine.

The results of the molecular docking study of four compounds with the highest biological activities (compounds **2, 4, 5,** and **7**) and naringenin on AChE are given in Table 4, Figures 1 and 2, and in the Supplementary Materials (Figures S1–S4).

The results indicated that studied substances were docked into the binding pocket of AChE with the same direction as the cocrystallized ligand (galantamine) in the protein complex. Derivatives **2, 5,** and **7** and naringenin all interacted with Glu199 and Ser200 at varying degrees, and these interactions were weaker than those produced by galantamine (hydrogen bonds with Glu199 (score: 63%, length 2.65 Å) and with Ser200 (score: 53%, length: 2.80 Å)). This may explain why the AChE inhibitory activities of these derivatives were weaker than that of galantamine. Substance **2** formed hydrogen bonds with both Glu199 (score: 25%, length 2.02 Å) and Ser200 (score: 19%, length: 3.01 Å); while derivatives **5** and **7** only made hydrogen bonds with Ser200 (compound **5**: score: 11%, length 3.02 Å; score: 16%, length: 2.87 Å; compound **7**: score: 31%, length 2.90 Å) and van der Waals interaction with Glu199; meanwhile, AChE inhibitory activities were in order **2** **<** **5<7**. This may suggest that a strong interaction with Ser200 is crucial for the inhibition of AChE. Substance **4** only made a weak interaction with Ser200 (van der Waals interaction) but had a stronger biological activity than derivatives **2** and **5**. This can be explained by the fact that derivative **4** had strong interactions with amino acid residues in the periphery region, which were hydrogen bonds with Tyr70 (score: 56%, length: 2.05 Å) and Tyr121 (score: 69%, length 2.54 Å). This also suggests an important role of peripheral interaction for the enzyme inhibitory activities of the compounds. Substance **7** made van der Waals interactions with 4/5 amino acids of the peripheral region (Asp72, Tyr121, Trp279, and Phe331); this contribution also explained partially the highest biological activity of derivative **7** in the studied compounds. Derivatives **2, 4, 5,** and **7**, naringenin, and galantamine all interacted with His440 (an amino acid of the catalytic triad). In addition to hydrogen bonding with His440 (score: 20%, length 2.00 Å), naringenin could only make van der Waals interactions with two amino acids Glu199 and Ser200 and with two amino acids Asp72 and Tyr121 of the peripheral region. This may partially explain why the AChE inhibitory activity of naringenin is weaker than that of derivatives **2, 4, 5,** and **7.**

### 3.4. Prediction Physicochemical Properties, Pharmacokinetics, and Toxicities

Predicted physicochemical properties, pharmacokinetics, and toxicities of synthesized compounds and galantamine are reported in the Supplementary Materials (Tables S10 and S11). The results showed that compound **7** (the most potential AChE inhibitor in this study) was predicted to have high capacities of absorption from the gastrointestinal tract and crossing the blood–brain barrier. This derivative was also expected to inhibit CYP1A2, CYP2C9, and CYP3A4. Regarding drug-likeness, derivative **7** satisfied the filters of Lipinski, Ghose, and Egan. This substance also did not have any alert on pan-assay interference compounds (PAINS).

In terms of toxicity, compound **7** was predicted to have a low acute oral toxicity with LD50 of 3800 mg/kg (toxicity class of 5), which was much less toxic than galantamine (LD50 of 85 mg/kg and toxicity class of 3). The compound was expected with high probabilities (0.71–0.98) and not to have hepatotoxicity, mutagenicity, and cytotoxicity or not to be active on aryl hydrocarbon receptor, androgen receptor ligand binding domain, aromatase, estrogen receptor ligand binding domain, peroxisome proliferator-activated receptor gamma, nuclear factor (erythroid-derived 2)-like 2/antioxidant responsive element, heat shock factor response element, and ATPase family AAA domain-containing protein 5. The compound was also assumed not to have carcinogenicity or not to be active on mitochondrial membrane potential, phosphoprotein (tumor suppressor) p53 (probabilities of 0.50–0.68).

It was anticipated that substance **7** had immunotoxicity with a high probability (0.92). In addition, this derivative was also expected to be active on estrogen receptor alpha, but with a correspondingly low probability of 0.59.

Thus, in addition to having the most potential AChE inhibitory activity among synthetic derivatives, compound **7** was also expected to have good pharmacokinetic properties, drug-likeness, and low toxicities on most common targets. Therefore, this is a promising substance for further studies.

## 4. Conclusions

In this study, based on the Williamson method, nine flavonoid derivatives, including two completely new structures, were synthesized from naringenin. The AChE enzyme inhibitory activities of the compounds were determined. The results indicated that four substances with improved activities compared to naringenin were obtained, in which compound **7** was the strongest enzyme inhibitor in the synthesized derivatives (IC_50_ 13.0 ± 1.9 *μ*M) with promising predicted profile in physicochemical properties, pharmacokinetics, and toxicities. Two new substances had negligible biological activities. However, they should be used in further studies on other targets. The molecular docking revealed that strong interactions with amino acid residue Ser200 of the catalytic triad and those of the peripheral region are crucial for strong inhibitory activities against AChE.

## Figures and Tables

**Table 1:** Predicted parameters by SwissADME.

Classification	Parameters
Physicochemical properties	Formula, molecular weight, number of heavy atoms, number of aromatic heavy atoms, fraction Csp3, number of rotatable bonds, number of H-bond acceptors, number of H-bond donors, molar refractivity, and topological polar surface area (TPSA)
Lipophilicity	Log Po/w (iLOGP), log Po/w (XLOGP3), log Po/w (WLOGP), log Po/w (MLOGP), log Po/w (SILICOS-IT), and consensus log Po/w
Water solubility	Log S (ESOL), log S (Ali), and log S (SILICOS-IT)
Pharmacokinetics	GI absorption, BBB permeant, P-gp substrate, CYP1A2 inhibitor, CYP2C19 inhibitor, CYP2C9 inhibitor, CYP2D6 inhibitor, CYP3A4 inhibitor, and log Kp (skin permeation)
Drug-likeness	Lipinski, Ghose, Veber, Egan, Muegge, and bioavailability score
Medicinal chemistry	PAINS, Brenk, lead-likeness, and synthetic accessibility

GI, gastrointestinal; BBB, blood–brain barrier; PAINS, pan-assay interference compounds.

**Table 2:** Predicted parameters by ProTox-II.

Classification	Target
Oral toxicity	LD50, toxicity class
Organ toxicity	Hepatotoxicity
Toxicity end points	Carcinogenicity, immunotoxicity, mutagenicity, and cytotoxicity
Tox21-nuclear receptor signalling pathways	Aryl hydrocarbon receptor (AhR), androgen receptor (AR), androgen receptor ligand binding domain (AR-LBD), aromatase, estrogen receptor alpha (ER), estrogen receptor ligand binding domain (ER-LBD), and peroxisome proliferator-activated receptor gamma (PPAR-gamma)
Tox21-stress response pathways	Nuclear factor (erythroid-derived 2)-like 2/antioxidant responsive element (nrf2/ARE), heat shock factor response element (HSE), mitochondrial membrane potential (MMP), phosphoprotein (tumor suppressor) p53, and ATPase family AAA domain-containing protein 5 (ATAD5)

LD50, median lethal dose; Tox21, toxicology in the 21st century (a unique collaboration between several US federal agencies to develop new ways to rapidly test whether substances adversely affect human health).

**Table 3:** AChE inhibitory activities of flavonoid derivatives.

No.	Compound	IC50 (*μ*M)
1	**1**	>>100.0
2	**2**	75.0 ± 4.8
3	**3**	>>100.0
4	**4**	39.8 ± 4.4
5	**5**	48.4 ± 2.9
6	**6**	>>100.0
7	**7**	13.0 ± 1.9
8	**8**	>>100.0
9	**9**	>>100.0
10	Naringenin	149.6 ± 4.6
11	Galantamine	1.3 ± 0.2

**Table 4:** Molecular docking result of synthesized flavonoid derivatives on AChE (PDB ID: 1W6R).

No.	Compound	Docking score (kJ·mol^−1^)	Interactions
2	**2**	−21.87	Hydrogen bonds with Glu199 (score: 25%, length 2.02 Å) and Ser200 (score: 19%, length: 3.01 Å).
			Van der Waals interaction with Trp84, Gly119, Tyr121, Phe290, Phe330, Phe331, Tyr334, His440, Gly441, and Tyr442.

4	**4**	−22.40	Hydrogen bonds with Tyr70 (score: 56%, length: 2.05 Å) and Tyr121 (score: 69%, length 2.54 Å).
			Van der Waals interaction with Asp72, Trp84, Gly118, Gly119, Ser200, Trp233, Trp279, Phe288, Phe290, Phe330, Phe331, Tyr334, and His440

5	**5**	−18.86	Hydrogen bonds with Trp84 (score: 33%, length 1.80 Å), Tyr121 (score: 82%, length: 2.58 Å), and Ser200 (score: 11%, length 3.02 Å; score: 16%, length: 2.87 Å).
			Van der Waals interaction with Gln69, Gly117, Ser122, Gly123, Gly118, Gly119, Glu199, Phe290, Phe330, Phe331, and His440.

7	**7**	−13.30	Hydrogen bonds with Ser200 (score: 31%, length 2.90 Å).
			Van der Waals interaction with Asp72, Trp84, Gly117, Gly118, Gly119, Tyr121, Tyr130, Glu199, Trp279, Phe290, Phe330, Phe331, Tyr334, His440, Gly441, and Ile444.

10	**Naringenin**	−21.12	Hydrogen bonds with His440 (score: 20%, length 2.00 Å).
			Van der Waals interaction with Asp72, Trp84, Asn85, Gly118, Gly119, Tyr121, Ser122, Glu199, Ser200, Phe330, Gly441, and Tyr442.

Asn, asparagine; Asp, aspartate; Gln, glutamine; Glu, glutamate; Gly, glycine; His, histidine; Ile, isoleucine; Phe, phenylalanine; Ser, serine; Trp, tryptophan; Tyr, tyrosine.

## Data Availability

The data used to support the findings of this study are included within the article.

## Supplementary Materials

Supplementary Materials Tables S1–S9: spectral data of compounds 1–9. Figure S1: mode of ligand interaction of compound 2 with AChE. Figure S2: mode of ligand interaction of compound 4 with AChE. Figure S3: mode of ligand interaction of compound 5 with AChE. Figure S4: mode of ligand interaction of naringenin with AChE. Table S10: pharmacokinetics, drug-likeness, and medicinal chemistry evaluation of studied compounds by SwissADME online service. Table S11: predicted toxicities of studied compounds by ProTox-II web server.
