# Supplementary material for: Synthesis and Evaluation of the Acetylcholinesterase Inhibitory Activities of Some Flavonoids Derived from Naringenin
Source: ScientificWorldJournal. 2021 Nov 30;2021:4817900. doi: 10.1155/2021/4817900 (PMC8651387; doi:10.1155/2021/4817900)
Supplement: Supplementary Materials — Tables S1–S9: spectral data of compounds 1–9. Figure S1: mode of ligand interaction of compound 2 with AChE. Figure S2: mode of ligand interaction of compound 4 with AChE. Figure S3: mode of ligand interaction of compound 5 with AChE. Figure S4: mode of ligand interaction of naringenin with AChE. Table S10: pharmacokinetics, drug-likeness, and medicinal chemistry evaluation of studied compounds by SwissADME online service. Table S11: predicted toxicities of studied compounds by ProTox-II web server. [file 4817900.f1.zip › 4817900.f1/Figure S1. Mode of ligand interaction of compound 2 with AChE.pdf]

Figure S1. Mode of ligand interaction of compound 2 with AChE

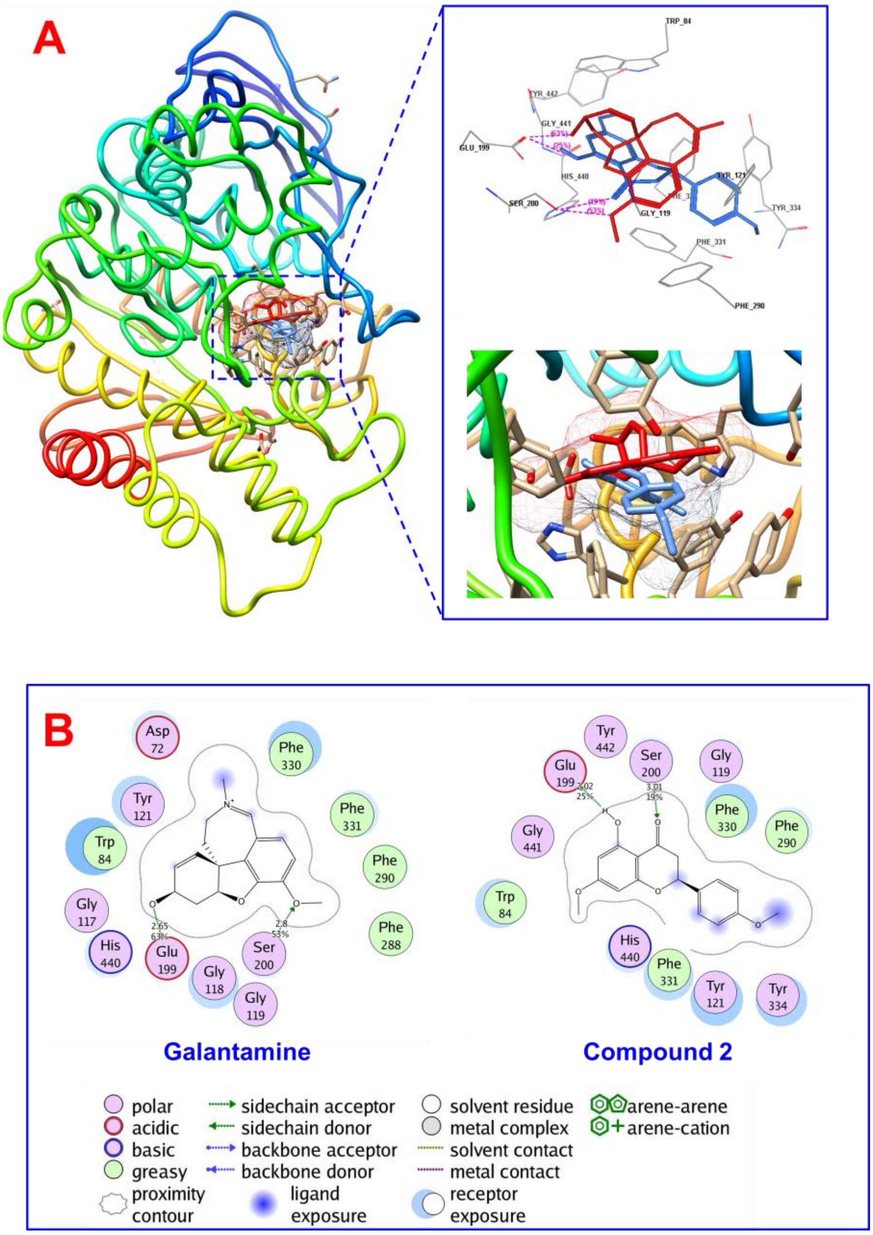

A. Alignment of docked pose of compound 2 with galantamine as co-crystallized ligand in the AChE complex (PDB ID: 1W6R). Compound 2 is rendered in cornflower blue, and galantamine is rendered in red.

B. Mode of interaction of galantamine and compound 2 with the enzyme AChE (depicted in two-dimensional format).

Asn: asparagine, Asp: aspartate, Gln: glutamine, Glu: glutamate, Gly: glycin, His: histidine, Il: isoleucine, Phe: phenylalanine, Ser: serine, Trp: tryptophan, Tyr: tyrosine.
