# Supplementary material for: Synthesis and Evaluation of the Acetylcholinesterase Inhibitory Activities of Some Flavonoids Derived from Naringenin
Source: ScientificWorldJournal. 2021 Nov 30;2021:4817900. doi: 10.1155/2021/4817900 (PMC8651387; doi:10.1155/2021/4817900)
Supplement: Supplementary Materials — Tables S1–S9: spectral data of compounds 1–9. Figure S1: mode of ligand interaction of compound 2 with AChE. Figure S2: mode of ligand interaction of compound 4 with AChE. Figure S3: mode of ligand interaction of compound 5 with AChE. Figure S4: mode of ligand interaction of naringenin with AChE. Table S10: pharmacokinetics, drug-likeness, and medicinal chemistry evaluation of studied compounds by SwissADME online service. Table S11: predicted toxicities of studied compounds by ProTox-II web server. [file 4817900.f1.zip › 4817900.f1/Table S1. Spectral data of compound 1.pdf]

# Table S1. Spectral data of compound 1

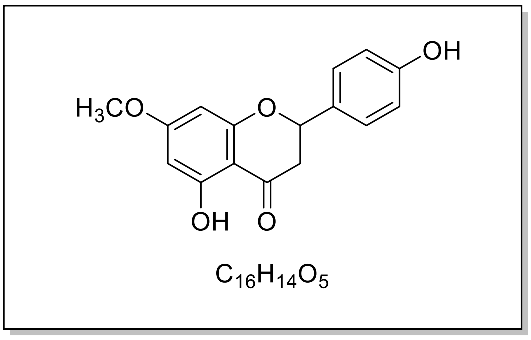


## UV

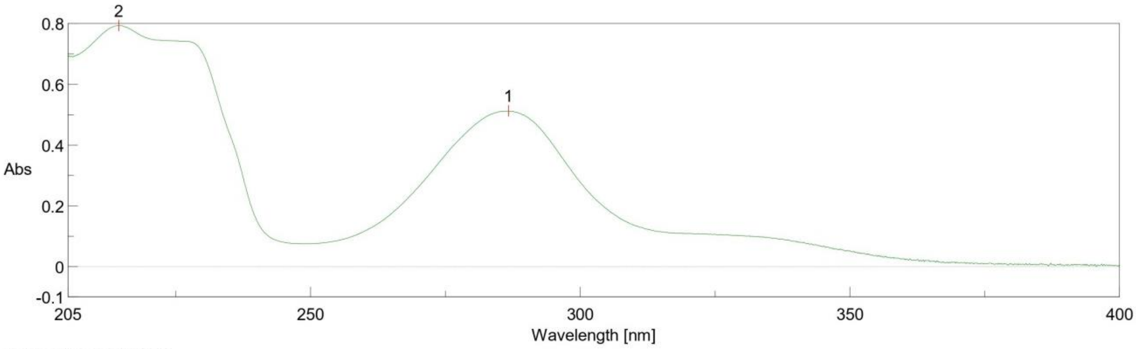


[ Result of Peak Picking ]

| No. | Position | Intensity | No. | Position | Intensity | No. | Position | Intensity |
|---|---|---|---|---|---|---|---|---|
| 1 | 286.7 | 0.512171 | 2 | 214.4 | 0.793113 | | | |

## IR

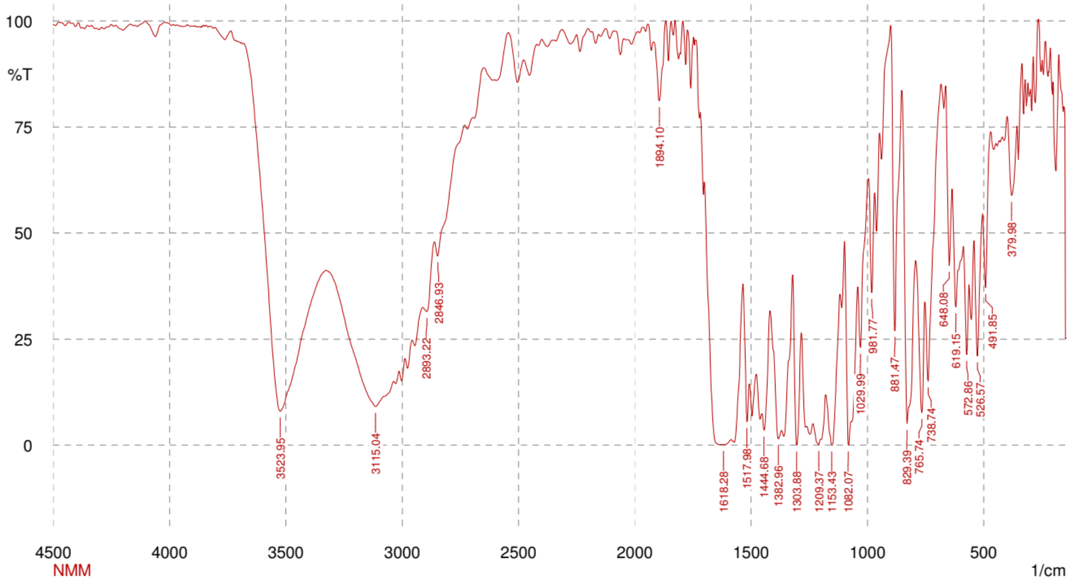

# HR-MS

## Qualitative Analysis Report

| | | | |
|---|---|---|---|
| **Data File** | NMM.d | **Sample Name** | NMM |
| **Sample Type** | Sample | **Position** | P1-A1 |
| **Instrument Name** | Instrument 1 | **User Name** | |
| **Acq Method** | Cot ngan - MSMS_Pos.m | **Acquired Time** | 01/07/2021 3:01:03 PM |
| **IRM Calibration Status** | Success | **DA Method** | COTNGAN.M.m |
| **Comment** | | | |
| **Sample Group** | | **Info.** | |
| **Stream Name** | LC 1 | **Acquisition SW Version** | 6200 series TOF/6500 series Q-TOF B.06.01 (B6172 SP1) |

**User Chromatograms**

| **Fragmentor Voltage** | 140 | **Collision Energy** | 0 | **Ionization Mode** | ESI |
|---|---|---|---|---|---|

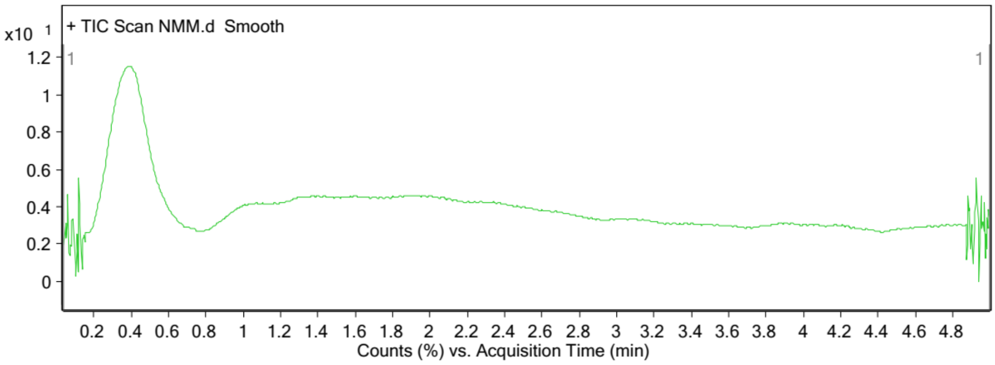




## Qualitative Analysis Report

**User Spectra**

| **Fragmentor Voltage** | **Collision Energy** | **Ionization Mode** |
|---|---|---|
| 140 | 0 | ESI |

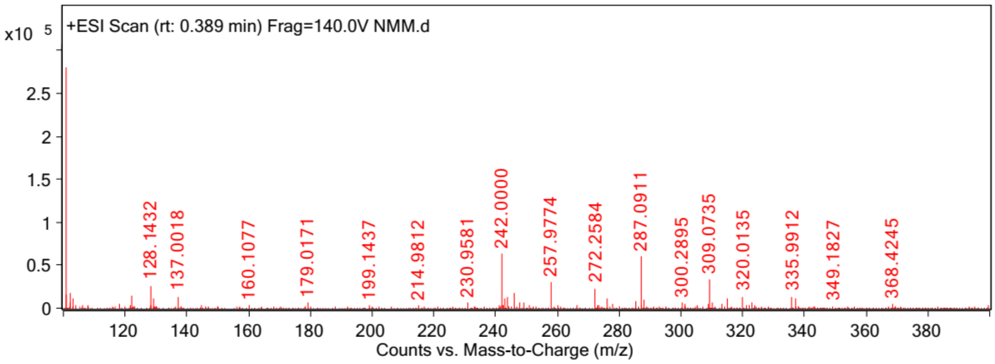

| | | | | | |
|---|---|---|---|---|---|
| **Sample Name** | NMM | **Position** | P1-A1 | **Instrument Name** | Instrument 1 |
| **User Name** | | **Inj Vol** | 2 | **InjPosition** | |
| **Sample Type** | Sample | **IRM Calibration Status** | Success | **Data Filename** | NMM.d |
| **ACQ Method** | Cot ngan - MSMS_Pos.m | **Comment** | | **Acquired Time** | 01/07/2021 3:01:03 PM |

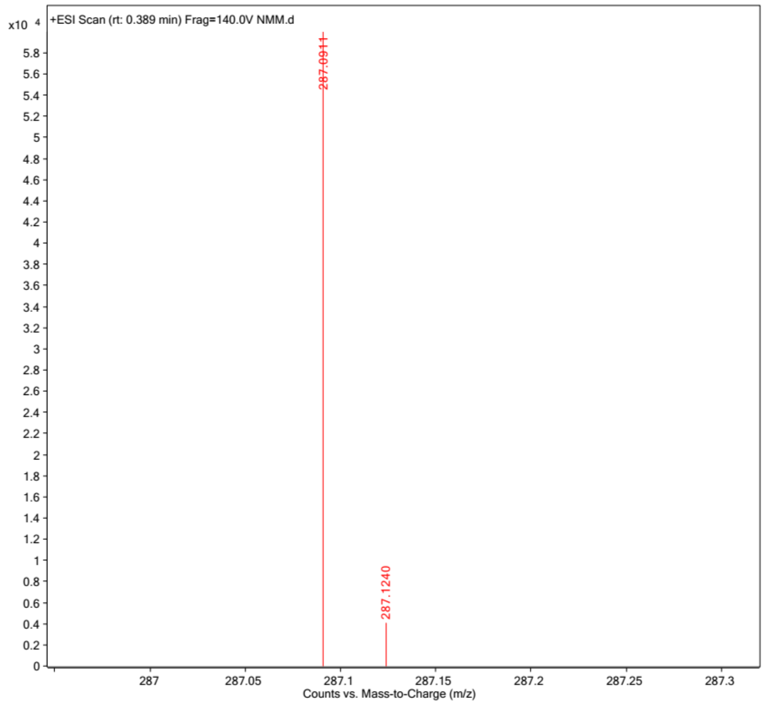

# $^{1}$H-NMR

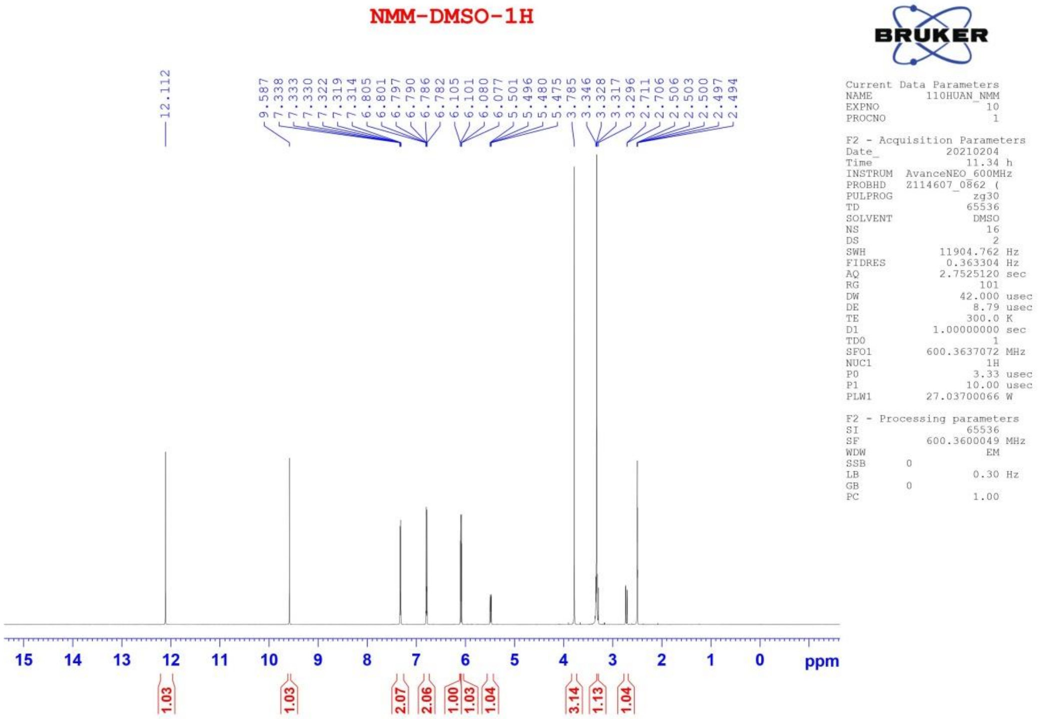
NMM-DMSO-1H
BRUKER
12.112
9.587
7.338
7.333
7.330
7.322
7.319
7.314
6.805
6.801
6.797
6.790
6.786
6.782
6.105
6.101
6.080
6.077
5.501
5.496
5.480
5.475
3.785
3.346
3.328
3.317
3.296
2.711
2.706
2.506
2.503
2.500
2.497
2.494
Current Data Parameters
NAME 110HUAN_NMM
EXPNO 10
PROCNO 1
F2 - Acquisition Parameters
Date_ 20210204
Time 11.34 h
INSTRUM AvanceNEO_600MHz
PROBHD Z114607_0862 (
PULPROG zg30
TD 65536
SOLVENT DMSO
NS 16
DS 2
SWH 11904.762 Hz
FIDRES 0.363304 Hz
AQ 2.7525120 sec
RG 101
DW 42.000 usec
DE 8.79 usec
TE 300.0 K
D1 1.00000000 sec
TD0 1
SFO1 600.3637072 MHz
NUC1 1H
P0 3.33 usec
P1 10.00 usec
PLW1 27.03700066 W
F2 - Processing parameters
SI 65536
SF 600.3600049 MHz
WDW EM
SSB 0
LB 0.30 Hz
GB 0
PC 1.00
15 14 13 12 11 10 9 8 7 6 5 4 3 2 1 0 ppm
1.03
1.03
2.07
2.06
1.00
1.03
1.04
3.14
1.13
1.04

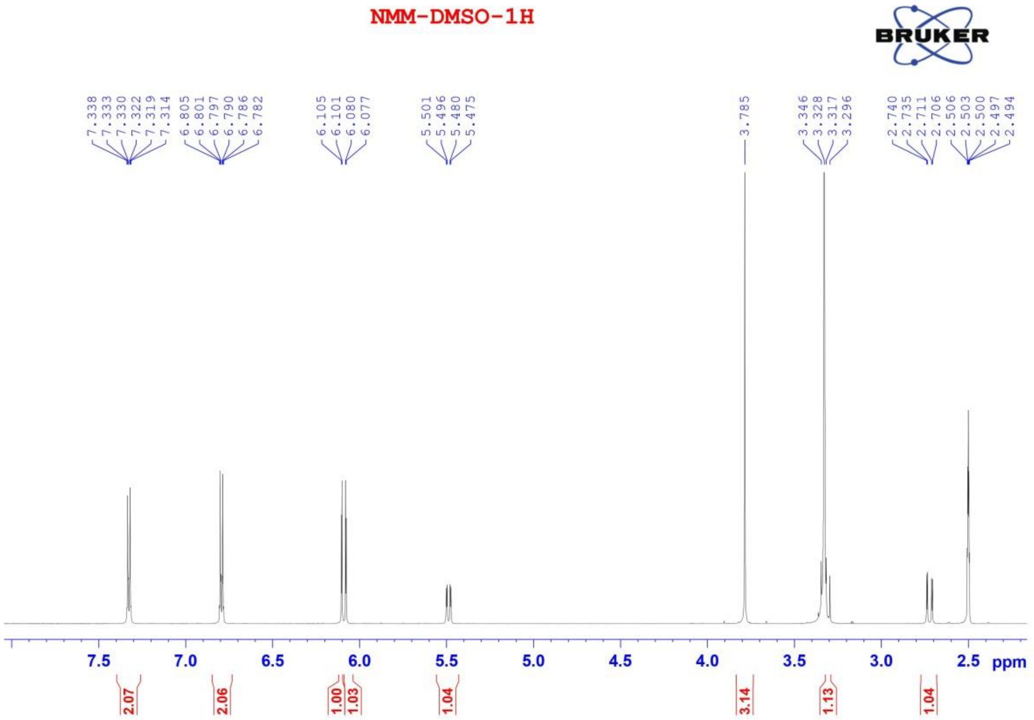
NMM-DMSO-1H
BRUKER
7.338
7.333
7.330
7.322
7.319
7.314
6.805
6.801
6.797
6.790
6.786
6.782
6.105
6.101
6.080
6.077
5.501
5.496
5.480
5.475
3.785
3.346
3.328
3.317
3.296
2.740
2.735
2.711
2.706
2.506
2.503
2.500
2.497
2.494
7.5 7.0 6.5 6.0 5.5 5.0 4.5 4.0 3.5 3.0 2.5 ppm
2.07
2.06
1.00
1.03
1.04
3.14
1.13
1.04

# $^{13}$C-NMR

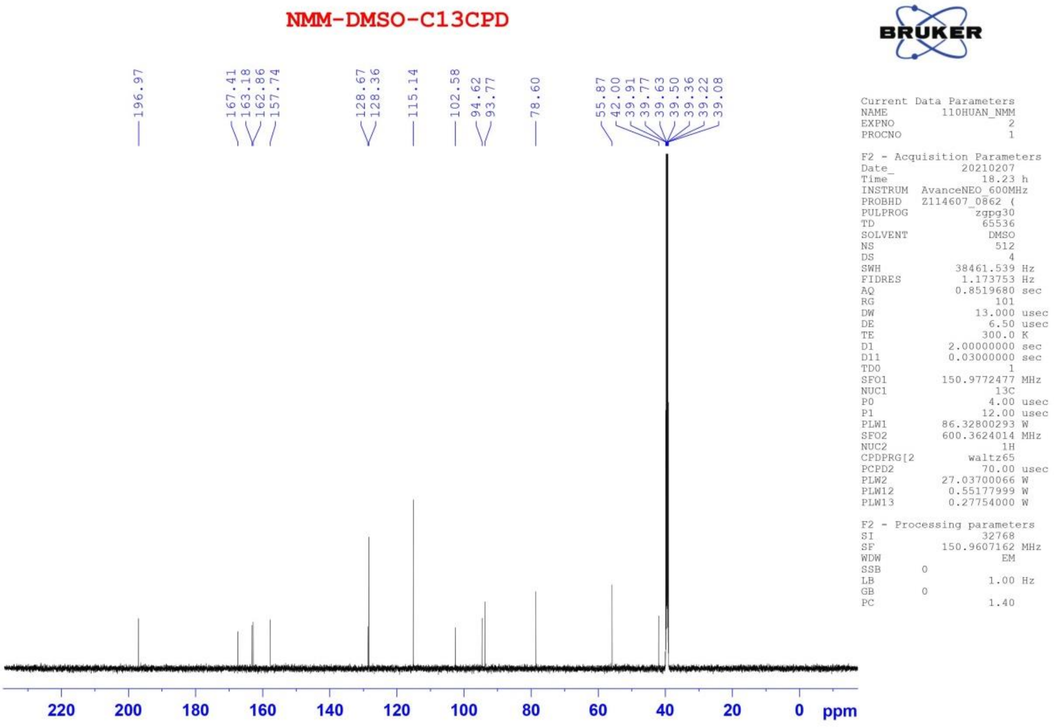
NMM-DMSO-C13CPD
BRUKER
196.97
167.41
163.18
162.86
157.74
128.67
128.36
115.14
102.58
94.62
93.77
78.60
55.87
42.00
39.91
39.77
39.63
39.50
39.36
39.22
39.08
Current Data Parameters
NAME 110HUAN_NMM
EXPNO 2
PROCNO 1
F2 - Acquisition Parameters
Date_ 20210207
Time 18.23 h
INSTRUM AvanceNEO_600MHz
PROBHD Z114607_0862 (
PULPROG zgpg30
TD 65536
SOLVENT DMSO
NS 512
DS 4
SWH 38461.539 Hz
FIDRES 1.173753 Hz
AQ 0.8519680 sec
RG 101
DW 13.000 usec
DE 6.50 usec
TE 300.0 K
D1 2.00000000 sec
D11 0.03000000 sec
TD0 1
SFO1 150.9772477 MHz
NUC1 13C
P0 4.00 usec
P1 12.00 usec
PLW1 86.32800293 W
SFO2 600.3624014 MHz
NUC2 1H
CPDPRG[2 waltz65
PCPD2 70.00 usec
PLW2 27.03700066 W
PLW12 0.55177999 W
PLW13 0.27754000 W
F2 - Processing parameters
SI 32768
SF 150.9607162 MHz
WDW EM
SSB 0
LB 1.00 Hz
GB 0
PC 1.40
220
200
180
160
140
120
100
80
60
40
20
0
ppm

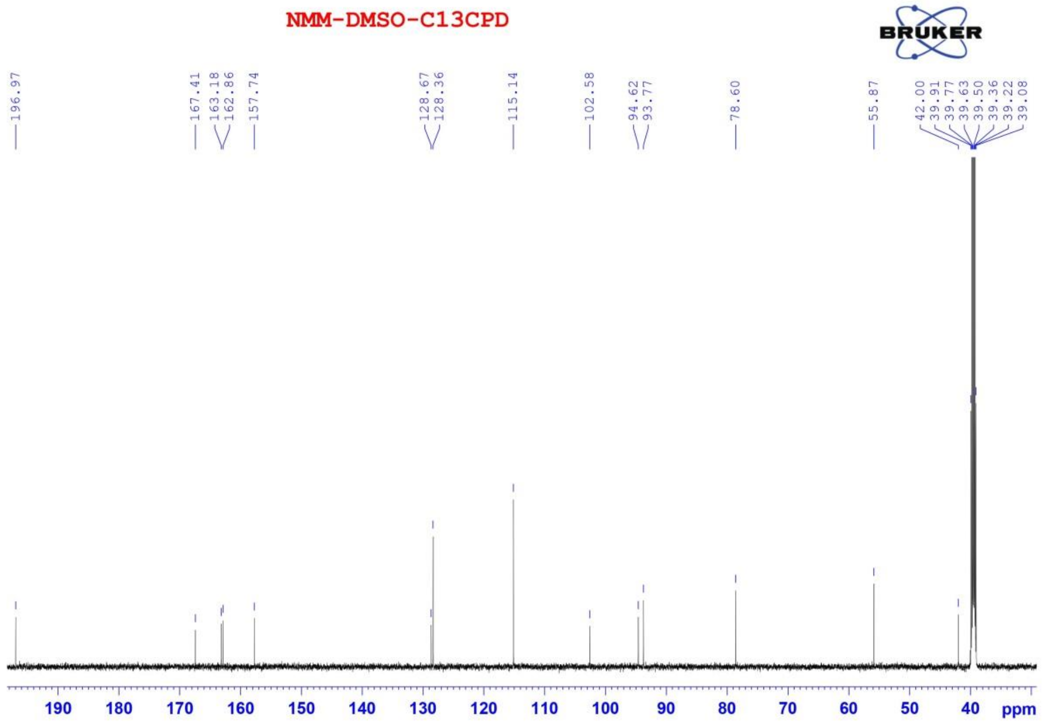
NMM-DMSO-C13CPD
BRUKER
196.97
167.41
163.18
162.86
157.74
128.67
128.36
115.14
102.58
94.62
93.77
78.60
55.87
42.00
39.91
39.77
39.63
39.50
39.36
39.22
39.08
190
180
170
160
150
140
130
120
110
100
90
80
70
60
50
40
ppm
