# Supplementary material for: Synthesis and Evaluation of the Acetylcholinesterase Inhibitory Activities of Some Flavonoids Derived from Naringenin
Source: ScientificWorldJournal. 2021 Nov 30;2021:4817900. doi: 10.1155/2021/4817900 (PMC8651387; doi:10.1155/2021/4817900)
Supplement: Supplementary Materials — Tables S1–S9: spectral data of compounds 1–9. Figure S1: mode of ligand interaction of compound 2 with AChE. Figure S2: mode of ligand interaction of compound 4 with AChE. Figure S3: mode of ligand interaction of compound 5 with AChE. Figure S4: mode of ligand interaction of naringenin with AChE. Table S10: pharmacokinetics, drug-likeness, and medicinal chemistry evaluation of studied compounds by SwissADME online service. Table S11: predicted toxicities of studied compounds by ProTox-II web server. [file 4817900.f1.zip › 4817900.f1/Table S10. Pharmacokinetics, drug-likeness and medicinal chemistry evaluation of studied compounds by SwissADME online service..pdf]

Table S10. Pharmacokinetics, drug-likeness and medicinal chemistry evaluation of studied compounds by SwissADME online service

Compound	1	2	3	4	5	6	7	8	9	GAL
#Heavy atoms	21	22	24	26	23	26	29	41	48	21
#Aromatic heavy atoms	12	12	12	12	12	12	12	30	36	6
Fraction Csp3	0.19	0.24	0.21	0.29	0.17	0.19	0.12	0.08	0.09	0.53
#Rotatable bonds	2	3	7	9	4	7	12	12	15	1
#H-bond acceptors	5	5	5	5	5	5	5	5	5	4
#H-bond donors	2	1	0	1	2	1	1	1	0	1
MR	76.04	80.51	92.22	102.17	85.18	98.79	115.17	161.21	190.16	84.05
TPSA	75.99	64.99	53.99	64.99	75.99	64.99	64.99	64.99	53.99	41.93
iLOGP	2.41	2.36	3.49	3.71	2.61	3.74	3.82	4.3	5.56	2.66
XLOGP3	2.85	3.17	3.58	4.9	3.49	4.46	5.73	8.28	9.55	1.84
WLOGP	2.49	2.79	3.51	4.38	3.04	3.9	4.87	7.46	9.18	1.32
MLOGP	0.96	1.2	1.99	2.44	1.36	1.97	2.86	4.75	5.76	1.74
Silicos-IT Log P	2.57	3.09	4.15	4.81	3.31	4.62	6.01	8.07	10.07	2.03
Consensus Log P	2.25	2.52	3.34	4.05	2.76	3.74	4.66	6.57	8.03	1.92
ESOL Log S	-3.7	-3.9	-4.04	-4.88	-4.1	-4.71	-5.4	-8.17	-9.34	-2.93
ESOL Solubility (mg/ml)	5.70E-02	3.74E-02	3.00E-02	4.65E-03	2.50E-02	6.81E-03	1.57E-03	3.67E-06	2.86E-07	3.41E-01
ESOL Solubility (mol/l)	1.99E-04	1.25E-04	9.14E-05	1.31E-05	8.00E-05	1.93E-05	4.01E-06	6.76E-09	4.52E-10	1.19E-03
ESOL Class	Soluble	Soluble	Moderately soluble	Moderately soluble	Moderately soluble	Moderately soluble	Moderately soluble	Poorly soluble	Poorly soluble	Soluble
Ali Log S	-4.1	-4.21	-4.4	-6	-4.77	-5.54	-6.86	-9.51	-10.6	-2.34
Ali Solubility (mg/ml)	2.25E-02	1.87E-02	1.31E-02	3.56E-04	5.32E-03	1.01E-03	5.39E-05	1.68E-07	1.61E-08	1.31E+00
Ali Solubility (mol/l)	7.86E-05	6.23E-05	3.98E-05	9.98E-07	1.70E-05	2.86E-06	1.37E-07	3.10E-10	2.54E-11	4.56E-03
Ali Class	Moderately soluble	Moderately soluble	Moderately soluble	Poorly soluble	Moderately soluble	Moderately soluble	Poorly soluble	Poorly soluble	Insoluble	Soluble
Silicos-IT LogSw	-4.12	-4.81	-5.44	-5.93	-4.57	-5.71	-6.08	-12.13	-15.25	-2.96
Silicos-IT Solubility (mg/ml)	2.19E-02	4.62E-03	1.20E-03	4.17E-04	8.44E-03	6.87E-04	3.26E-04	3.98E-10	3.53E-13	3.17E-01
Silicos-IT Solubility (mol/l)	7.66E-05	1.54E-05	3.65E-06	1.17E-06	2.70E-05	1.95E-06	8.30E-07	7.33E-13	5.57E-16	1.10E-03
Silicos-IT class	Moderately soluble	Moderately soluble	Moderately soluble	Moderately soluble	Moderately soluble	Moderately soluble	Poorly soluble	Insoluble	Insoluble	Soluble
GI absorption	High	High	High	High	High	High	High	Low	Low	High
BBB permeant	Yes	Yes	Yes	Yes	Yes	Yes	Yes	No	No	Yes

Compound	1	2	3	4	5	6	7	8	9	GAL
Pgp substrate	No	No	No	No	No	No	No	Yes	Yes	Yes
CYP1A2 inhibitor	Yes	Yes	Yes	Yes	Yes	Yes	Yes	No	No	No
CYP2C19 inhibitor	Yes	Yes	Yes	Yes	Yes	Yes	No	No	No	No
CYP2C9 inhibitor	No	No	Yes	Yes	Yes	Yes	Yes	No	No	No
CYP2D6 inhibitor	No	Yes	Yes	Yes	Yes	Yes	No	Yes	No	Yes
CYP3A4 inhibitor	Yes	Yes	Yes	Yes	Yes	Yes	Yes	No	No	No
log Kp (cm/s)	-6.02	-5.88	-5.76	-5	-5.73	-5.28	-4.63	-3.73	-3.38	-6.75
Lipinski #violations	0	0	0	0	0	0	0	2	2	0
Ghose #violations	0	0	0	0	0	0	0	4	4	0
Veber #violations	0	0	0	0	0	0	1	1	1	0
Egan #violations	0	0	0	0	0	0	0	1	1	0
Muegge #violations	0	0	0	0	0	0	1	1	2	0
Bioavailability Score	0.55	0.55	0.55	0.55	0.55	0.55	0.55	0.17	0.17	0.55
PAINS #alerts	0	0	0	0	0	0	0	0	0	0
Brenk #alerts	0	0	1	1	1	1	2	1	1	1
Leadlikeness #violations	0	0	1	3	0	2	3	3	3	0
Synthetic Accessibility	3.11	3.22	2.98	3.3	3.29	3.54	3.46	4.17	4.69	4.57

#: number, GI: gastrointestinal, BBB: blood–brain barrier , PAINS: pan-assay interference compounds.
