# Supplementary material for: Synthesis and Evaluation of the Acetylcholinesterase Inhibitory Activities of Some Flavonoids Derived from Naringenin
Source: ScientificWorldJournal. 2021 Nov 30;2021:4817900. doi: 10.1155/2021/4817900 (PMC8651387; doi:10.1155/2021/4817900)
Supplement: Supplementary Materials — Tables S1–S9: spectral data of compounds 1–9. Figure S1: mode of ligand interaction of compound 2 with AChE. Figure S2: mode of ligand interaction of compound 4 with AChE. Figure S3: mode of ligand interaction of compound 5 with AChE. Figure S4: mode of ligand interaction of naringenin with AChE. Table S10: pharmacokinetics, drug-likeness, and medicinal chemistry evaluation of studied compounds by SwissADME online service. Table S11: predicted toxicities of studied compounds by ProTox-II web server. [file 4817900.f1.zip › 4817900.f1/Table S11. Predicted toxicities of studied compounds by Pro Tox-II web server.pdf]

Table S11. Predicted toxicities of studied compounds by Pro Tox-II web server.

Classification	Target	1		2		3		4		5		6		7		8		9		GAL	
		Pred	Prob	Pred	Prob	Pred	Prob	Pred	Prob	Pred	Prob	Pred	Prob	Pred	Prob	Pred	Prob	Pred	Prob	Pred	Prob
Organ toxicity	Hepatotoxicity	-	0.71	-	0.69	-	0.56	-	0.74	-	0.7	-	0.73	-	0.71	-	0.75	-	0.75	-	0.93
Toxicity end points	Carcinogenicity	-	0.52	-	0.5	-	0.61	-	0.67	-	0.58	-	0.53	-	0.67	-	0.66	-	0.6	-	0.65
Toxicity end points	Immunotoxicity	+	0.51	+	0.61	+	0.97	+	0.96	-	0.64	+	0.5	+	0.92	+	0.77	-	0.57	+	0.98
Toxicity end points	Mutagenicity	-	0.62	-	0.64	-	0.7	-	0.74	-	0.62	-	0.63	-	0.77	-	0.77	-	0.72	-	0.76
Toxicity end points	Cytotoxicity	-	0.71	-	0.8	-	0.64	-	0.94	-	0.7	-	0.71	-	0.91	-	0.87	-	0.89	+	0.5
Tox21-Nuclear receptor signalling pathways	Aryl hydrocarbon Receptor (AhR)	+	0.5	+	0.5	+	0.62	-	0.68	-	0.67	-	0.74	-	0.78	-	0.79	-	0.76	+	1
Tox21-Nuclear receptor signalling pathways	Androgen Receptor (AR)	-	0.96	-	0.96	-	0.98	-	0.98	-	0.83	-	0.78	-	0.92	-	0.95	-	0.95	-	0.9
Tox21-Nuclear receptor signalling pathways	Androgen Receptor Ligand Binding Domain (AR-LBD)	-	0.98	-	0.99	-	0.99	-	0.99	-	0.97	-	0.98	-	0.98	-	0.99	-	0.99	-	0.99
Tox21-Nuclear receptor signalling pathways	Aromatase	-	0.78	-	0.8	-	0.85	-	0.87	-	0.79	-	0.81	-	0.88	-	0.88	-	0.88	-	0.97
Tox21-Nuclear receptor signalling pathways	Estrogen Receptor Alpha (ER)	+	0.68	+	0.64	+	0.65	+	0.53	-	0.51	-	0.54	+	0.59	+	0.61	+	0.54	-	0.96

Classification	Target	1		2		3		4		5		6		7		8		9		GAL	
		**Pred**	**Prob**	**Pred**	**Prob**	**Pred**	**Prob**	**Pred**	**Prob**	**Pred**	**Prob**	**Pred**	**Prob**	**Pred**	**Prob**	**Pred**	**Prob**	**Pred**	**Prob**	**Pred**	**Prob**
Tox21-Nuclear receptor signalling pathways	Estrogen Receptor Ligand Binding Domain (ER-LBD)	+	0.56	-	0.52	-	0.74	-	0.74	-	0.78	-	0.85	-	0.76	-	0.74	-	0.9	-	0.99
Tox21-Nuclear receptor signalling pathways	Peroxisome Proliferator Activated Receptor Gamma (PPAR-Gamma)	-	0.71	-	0.75	-	0.93	-	0.87	-	0.73	-	0.79	-	0.87	-	0.9	-	0.93	-	1
Tox21-Stress response pathways	Nuclear factor (erythroid-derived 2)-like 2/antioxidant responsive element (nrf2/ARE)	-	0.88	-	0.89	+	1	-	0.57	-	0.87	-	0.83	-	0.72	-	0.74	-	0.7	-	0.93
Tox21-Stress response pathways	Heat shock factor response element (HSE)	-	0.88	-	0.89	+	1	-	0.57	-	0.87	-	0.83	-	0.72	-	0.74	-	0.7	-	0.93
Tox21-Stress response pathways	Mitochondrial Membrane Potential (MMP)	+	0.69	+	0.67	+	0.69	-	0.5	-	0.56	-	0.68	-	0.5	+	0.53	-	0.55	-	0.98
Tox21-Stress response pathways	Phosphoprotein (Tumor Suppressor) p53	-	0.72	-	0.85	-	0.76	-	0.66	-	0.7	-	0.8	-	0.68	-	0.67	-	0.73	-	0.97

Classification	Target	1		2		3		4		5		6		7		8		9		GAL	
		**Pred**	**Prob**	**Pred**	**Prob**	**Pred**	**Prob**	**Pred**	**Prob**	**Pred**	**Prob**	**Pred**	**Prob**	**Pred**	**Prob**	**Pred**	**Prob**	**Pred**	**Prob**	**Pred**	**Prob**
Tox21-Stress response pathways	ATPase family AAA domain-containing protein 5 (ATAD5)	+	0.51	-	0.51	-	0.79	-	0.9	-	0.81	-	0.86	-	0.8	-	0.76	-	0.8	-	0.98
Acute Oral Toxicity	Predicted LD50 (mg/kg)	2000		2000		3000		3800		2000		2000		3800		3800		3800		85	
	Predicted Toxicity Class	4		4		5		5		4		4		5		5		5		3	

+: active, -: inactive, Pred: predicted, Prob: probability, GAL: galantamine, LD50: median lethal dose, Tox21: Toxicology in the 21st Century (a unique collaboration between several US federal agencies to develop new ways to rapidly test whether substances adversely affect human health).
