# Supplementary material for: Synthesis and Evaluation of the Acetylcholinesterase Inhibitory Activities of Some Flavonoids Derived from Naringenin
Source: ScientificWorldJournal. 2021 Nov 30;2021:4817900. doi: 10.1155/2021/4817900 (PMC8651387; doi:10.1155/2021/4817900)
Supplement: Supplementary Materials — Tables S1–S9: spectral data of compounds 1–9. Figure S1: mode of ligand interaction of compound 2 with AChE. Figure S2: mode of ligand interaction of compound 4 with AChE. Figure S3: mode of ligand interaction of compound 5 with AChE. Figure S4: mode of ligand interaction of naringenin with AChE. Table S10: pharmacokinetics, drug-likeness, and medicinal chemistry evaluation of studied compounds by SwissADME online service. Table S11: predicted toxicities of studied compounds by ProTox-II web server. [file 4817900.f1.zip › 4817900.f1/Table S2. Spectral data of compound 2.pdf]

Table S2. Spectral data of compound 2

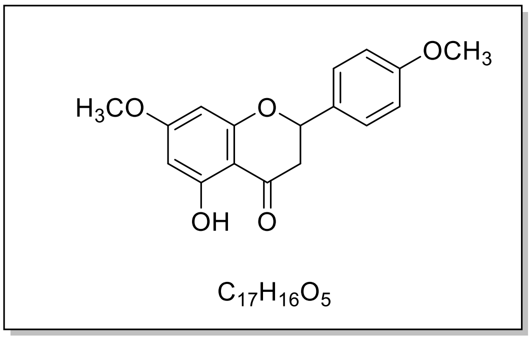

UV

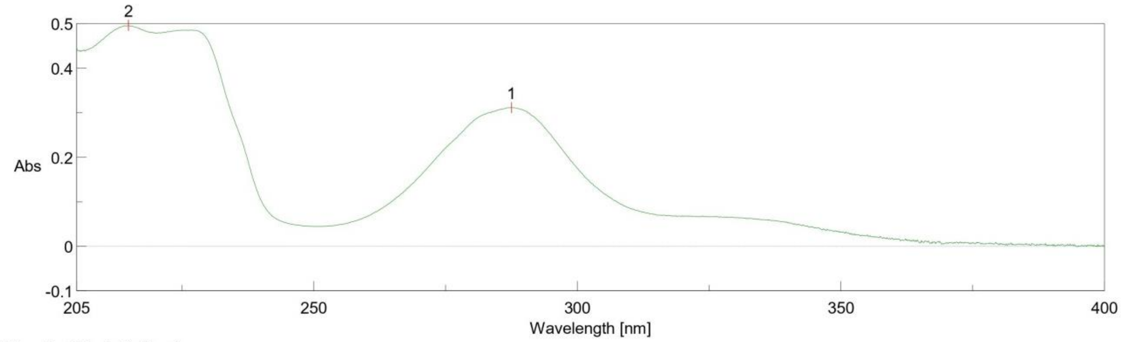

[Result of Peak Picking]

No.	Position	Intensity	No.	Position	Intensity	No.	Position	Intensity
1	287.5	0.311437	2	214.8	0.496255			

IR

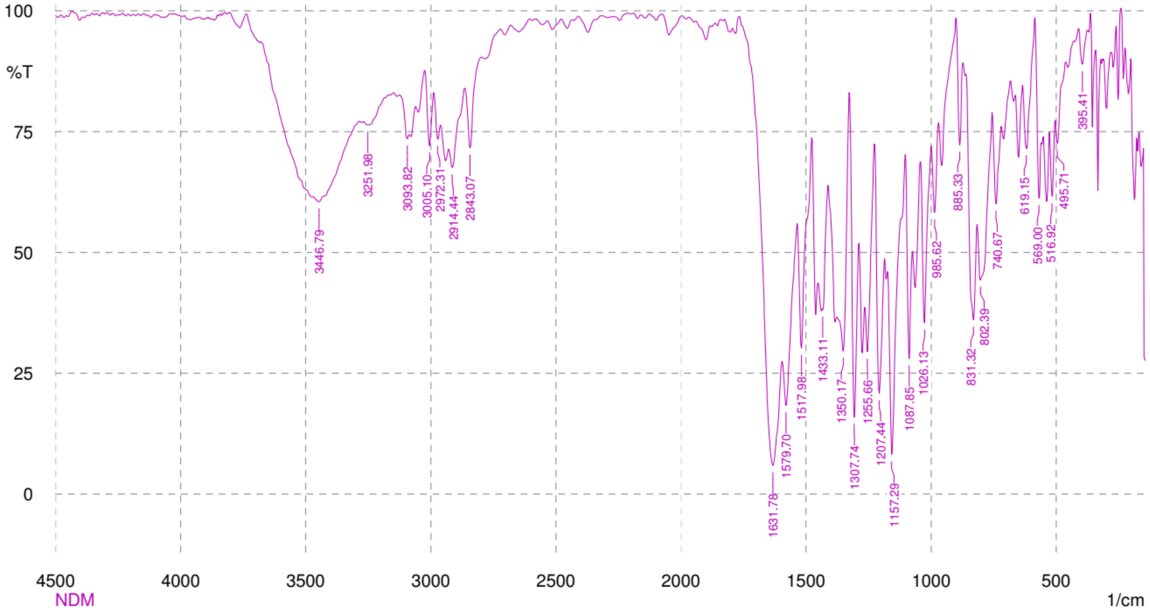

HR-MS

Qualitative Analysis Report

**Data File**	NDM.d	**Sample Name**	NDM
**Sample Type**	Sample	**Position**	P1-A2
**Instrument Name**	Instrument 1	**User Name**	
**Acq Method**	Cot ngan - MSMS_Pos.m	**Acquired Time**	01/07/2021 3:06:33 PM
**IRM Calibration Status**	Success	**DA Method**	COTNGAN.M.m
**Comment**			
**Sample Group**		**Info.**	
**Stream Name**	LC 1	**Acquisition SW Version**	6200 series TOF/6500 series Q-TOF B.06.01 (B6172 SP1)

User Chromatograms

**Fragmentor Voltage**	140	**Collision Energy**	0	**Ionization Mode**	ESI

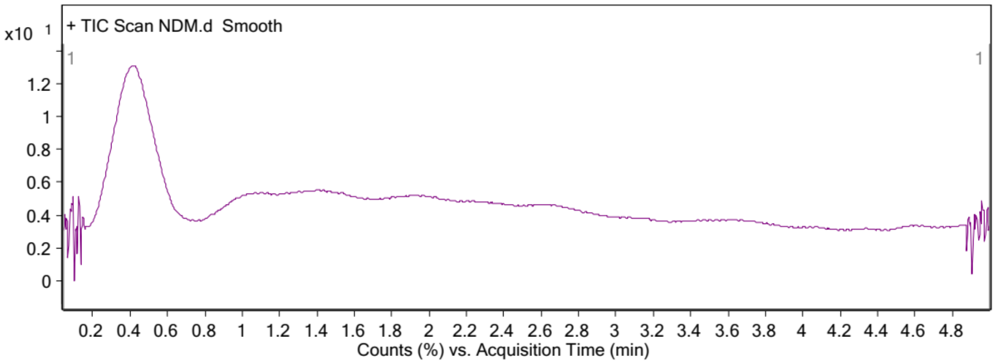

Qualitative Analysis Report

User Spectra

**Fragmentor Voltage**	**Collision Energy**	**Ionization Mode**
140	0	ESI

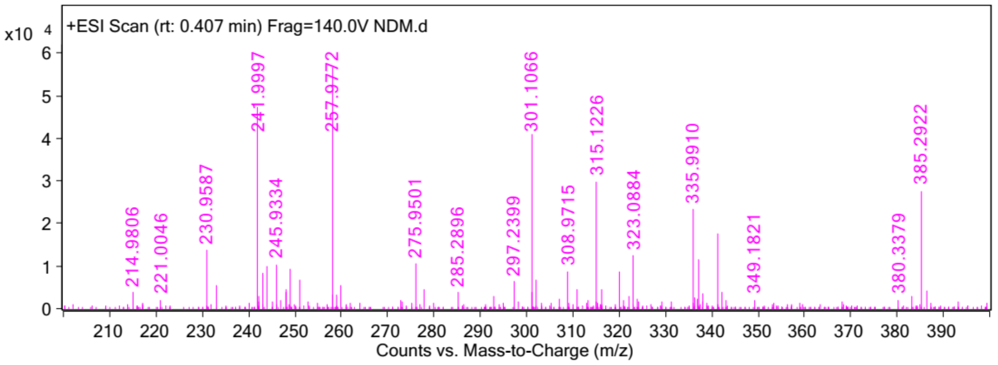

**Sample Name**	NDM	**Position**	P1-A2	**Instrument Name**	Instrument 1
**User Name**		**Inj Vol**	2	**InjPosition**	
**Sample Type**	Sample	**IRM Calibration Status**	Success	**Data Filename**	NDM.d
**ACQ Method**	Cot ngan - MSMS_Pos.m	**Comment**		**Acquired Time**	01/07/2021 3:06:33 PM

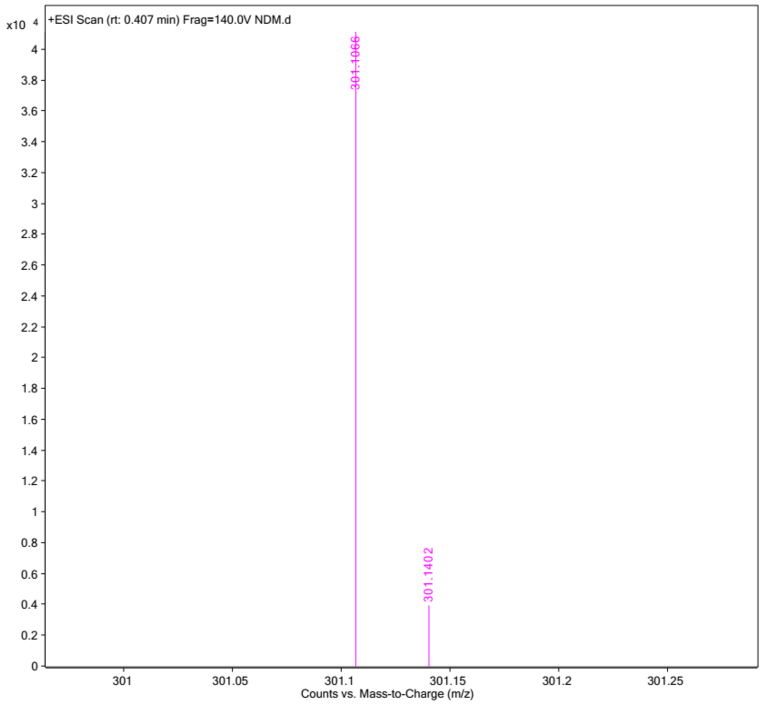

^{1}H-NMR

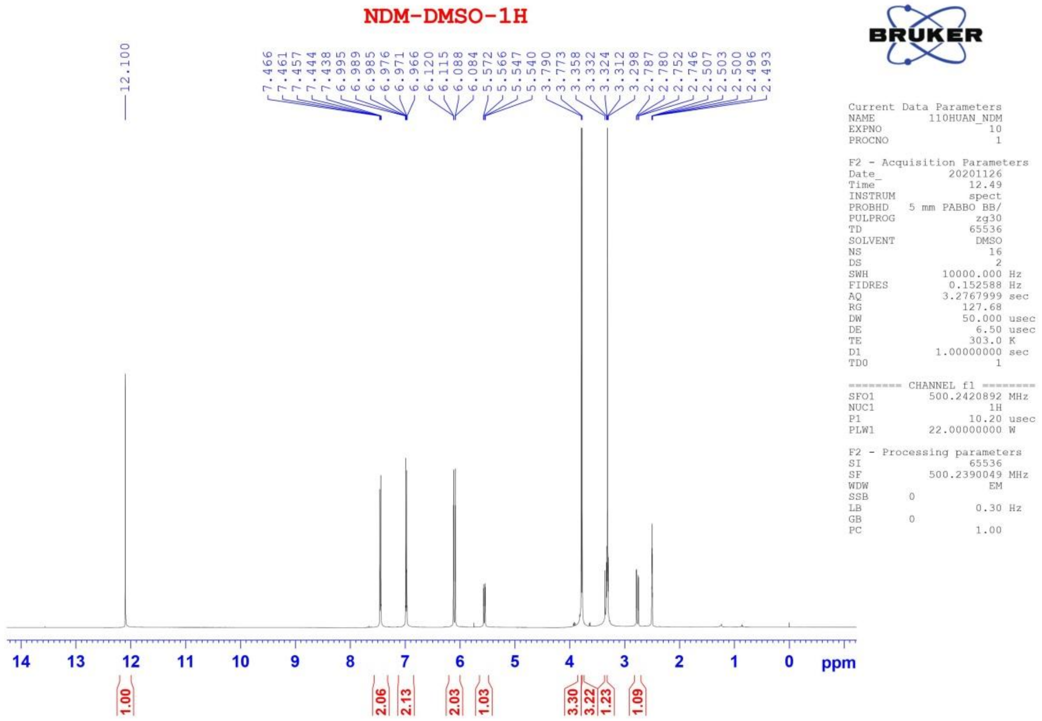
NDM-DMSO-1H
12.100
7.466 7.461 7.457 7.444 7.438 6.995 6.989 6.985 6.976 6.971 6.966 6.120 6.115 6.088 6.084 5.572 5.566 5.547 5.540 3.790 3.773 3.358 3.332 3.324 3.312 3.298 2.787 2.780 2.752 2.746 2.507 2.503 2.500 2.496 2.493
Current Data Parameters
NAME 110HUAN_NDM
EXPNO 10
PROCNO 1
F2 - Acquisition Parameters
Date_ 20201126
Time 12.49
INSTRUM spect
PROBHD 5 mm PABBO BB/
PULPROG zg30
TD 65536
SOLVENT DMSO
NS 16
DS 2
SWH 10000.000 Hz
FIDRES 0.152588 Hz
AQ 3.2767999 sec
RG 127.68
DW 50.000 usec
DE 6.50 usec
TE 303.0 K
D1 1.00000000 sec
TD0 1
======== CHANNEL f1 ========
SFO1 500.2420892 MHz
NUC1 1H
P1 10.20 usec
PLW1 22.00000000 W
F2 - Processing parameters
SI 65536
SF 500.2390049 MHz
WDW EM
SSB 0
LB 0.30 Hz
GB 0
PC 1.00
14 13 12 11 10 9 8 7 6 5 4 3 2 1 0 ppm
1.00 2.06 2.13 2.03 1.03 3.30 3.22 1.23 1.09

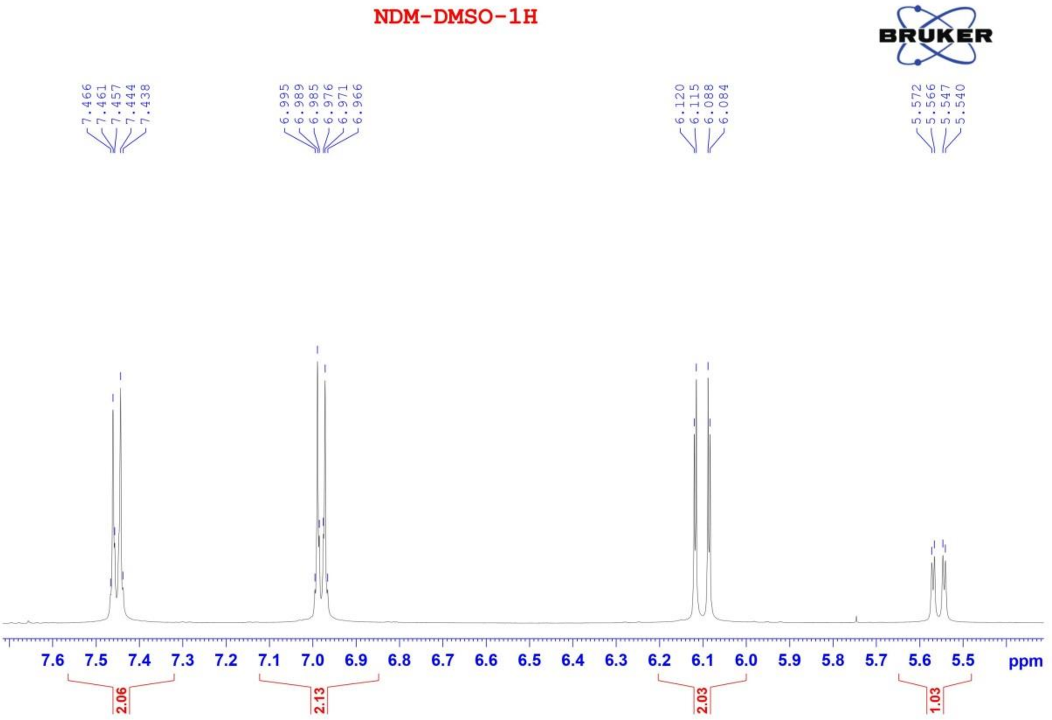
NDM-DMSO-1H
7.466 7.461 7.457 7.444 7.438
6.995 6.989 6.985 6.976 6.971 6.966
6.120 6.115 6.088 6.084
5.572 5.566 5.547 5.540
7.6 7.5 7.4 7.3 7.2 7.1 7.0 6.9 6.8 6.7 6.6 6.5 6.4 6.3 6.2 6.1 6.0 5.9 5.8 5.7 5.6 5.5 ppm
2.06 2.13 2.03 1.03

^{13}C-NMR

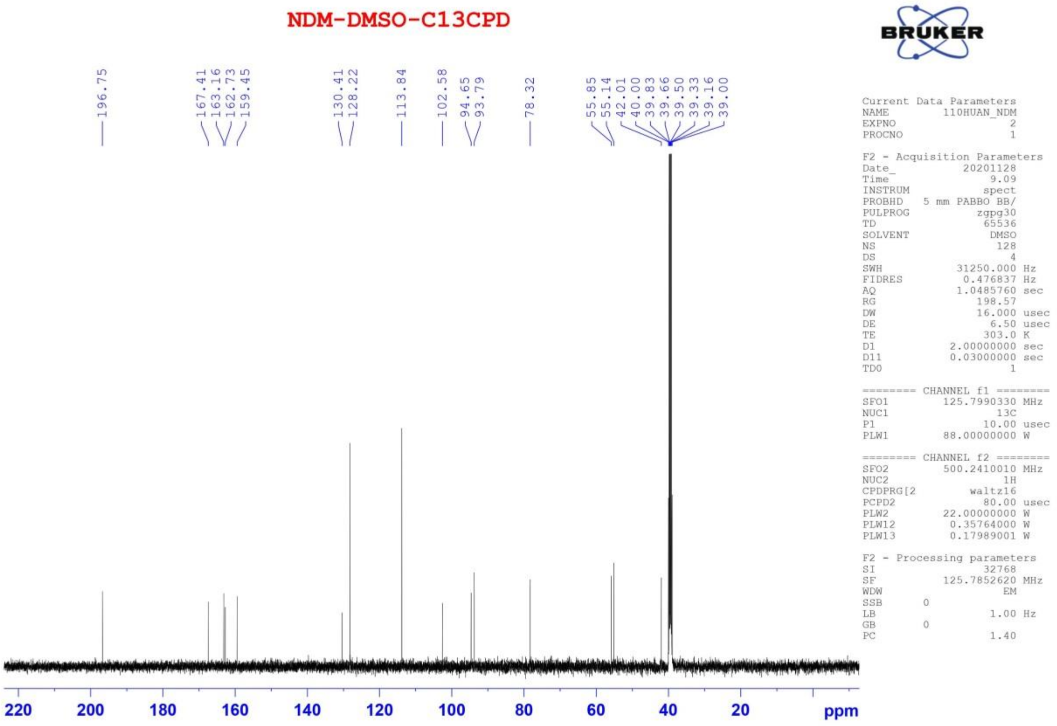
NDM-DMSO-C13CPD
BRUKER
196.75
167.41
163.16
162.73
159.45
130.41
128.22
113.84
102.58
94.65
93.79
78.32
55.85
55.14
42.01
40.00
39.83
39.66
39.50
39.33
39.16
39.00
Current Data Parameters
NAME 110HUAN_NDM
EXPNO 2
PROCNO 1
F2 - Acquisition Parameters
Date_ 20201128
Time 9.09
INSTRUM spect
PROBHD 5 mm PABBO BB/
PULPROG zgpg30
TD 65536
SOLVENT DMSO
NS 128
DS 4
SWH 31250.000 Hz
FIDRES 0.476837 Hz
AQ 1.0485760 sec
RG 198.57
DW 16.000 usec
DE 6.50 usec
TE 303.0 K
D1 2.00000000 sec
D11 0.03000000 sec
TD0 1
======== CHANNEL f1 ========
SFO1 125.7990330 MHz
NUC1 13C
P1 10.00 usec
PLW1 88.00000000 W
======== CHANNEL f2 ========
SFO2 500.2410010 MHz
NUC2 1H
CPDPRG[2 waltz16
PCPD2 80.00 usec
PLW2 22.00000000 W
PLW12 0.35764000 W
PLW13 0.17989001 W
F2 - Processing parameters
SI 32768
SF 125.7852620 MHz
WDW EM
SSB 0
LB 1.00 Hz
GB 0
PC 1.40
220
200
180
160
140
120
100
80
60
40
20
ppm

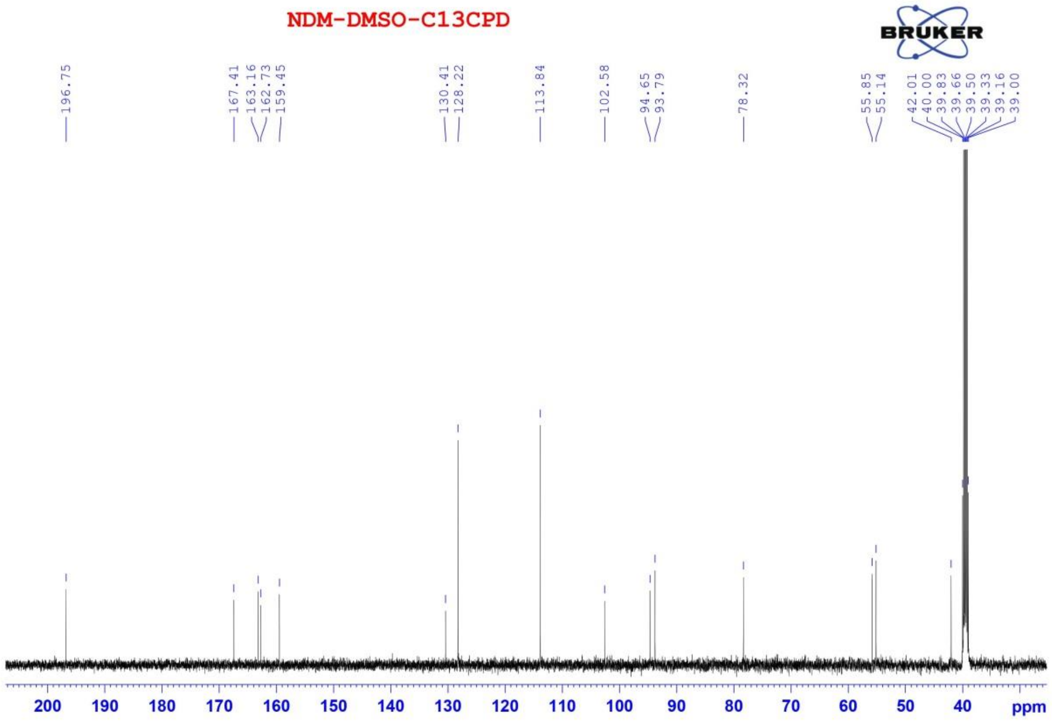
NDM-DMSO-C13CPD
BRUKER
196.75
167.41
163.16
162.73
159.45
130.41
128.22
113.84
102.58
94.65
93.79
78.32
55.85
55.14
42.01
40.00
39.83
39.66
39.50
39.33
39.16
39.00
200
190
180
170
160
150
140
130
120
110
100
90
80
70
60
50
40
ppm
