# Supplementary material for: Synthesis and Evaluation of the Acetylcholinesterase Inhibitory Activities of Some Flavonoids Derived from Naringenin
Source: ScientificWorldJournal. 2021 Nov 30;2021:4817900. doi: 10.1155/2021/4817900 (PMC8651387; doi:10.1155/2021/4817900)
Supplement: Supplementary Materials — Tables S1–S9: spectral data of compounds 1–9. Figure S1: mode of ligand interaction of compound 2 with AChE. Figure S2: mode of ligand interaction of compound 4 with AChE. Figure S3: mode of ligand interaction of compound 5 with AChE. Figure S4: mode of ligand interaction of naringenin with AChE. Table S10: pharmacokinetics, drug-likeness, and medicinal chemistry evaluation of studied compounds by SwissADME online service. Table S11: predicted toxicities of studied compounds by ProTox-II web server. [file 4817900.f1.zip › 4817900.f1/Table S6. Spectral data of compound 6.pdf]

Table S6. Spectral data of compound 6

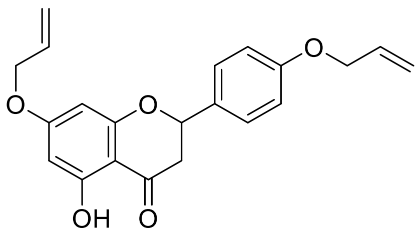

$C_{21}H_{20}O_5$

UV

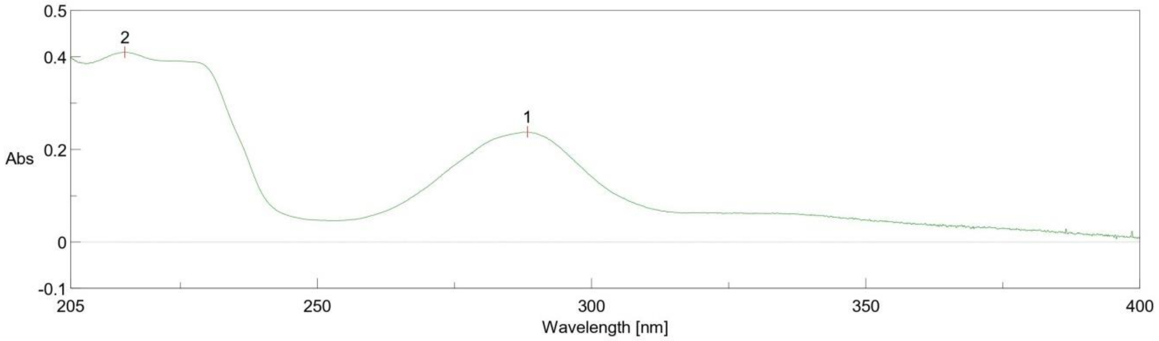

[Result of Peak Picking]

No.	Position	Intensity	No.	Position	Intensity	No.	Position	Intensity
1	288.3	0.237612	2	214.9	0.40992			

IR

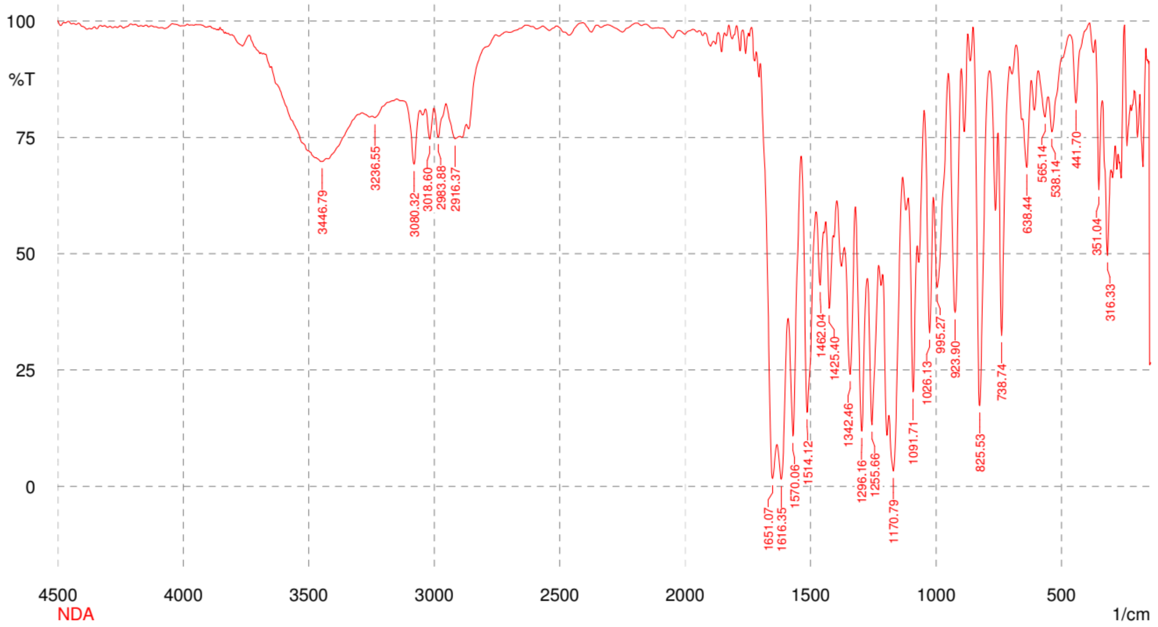

HR-MS

Qualitative Analysis Report

**Data File**	NDA.d	**Sample Name**	NDA
**Sample Type**	Sample	**Position**	P1-A6
**Instrument Name**	Instrument 1	**User Name**	
**Acq Method**	Cot ngan - MSMS_Pos.m	**Acquired Time**	01/07/2021 3:28:46 PM
**IRM Calibration Status**	Success	**DA Method**	COTNGAN.M.m
**Comment**			
**Sample Group**		**Info.**	
**Stream Name**	LC 1	**Acquisition SW Version**	6200 series TOF/6500 series Q-TOF B.06.01 (B6172 SP1)

User Chromatograms

**Fragmentor Voltage**	140	**Collision Energy**	0	**Ionization Mode**	ESI

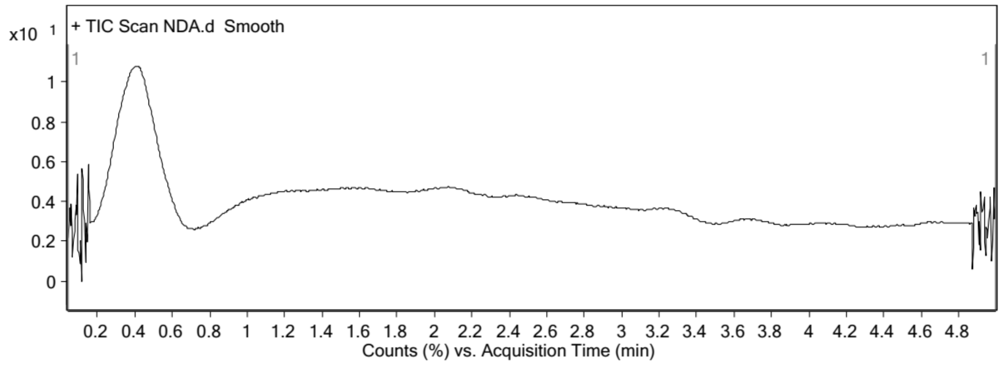

Qualitative Analysis Report

User Spectra

Fragmentor Voltage	Collision Energy	Ionization Mode
140	0	ESI

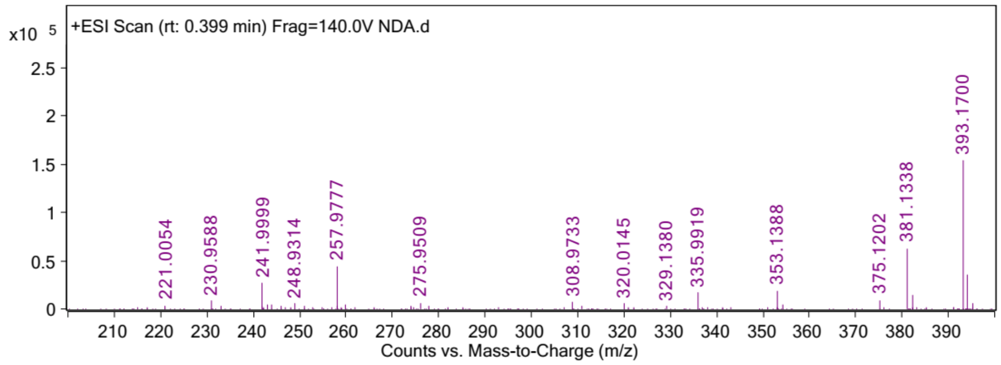

**Sample Name**	NDA	**Position**	P1-A6	**Instrument Name**	Instrument 1
**User Name**		**Inj Vol**	2	**InjPosition**	
**Sample Type**	Sample	**IRM Calibration Status**	Success	**Data Filename**	NDA.d
**ACQ Method**	Cot ngan - MSMS_Pos.m	**Comment**		**Acquired Time**	01/07/2021 3:28:46 PM

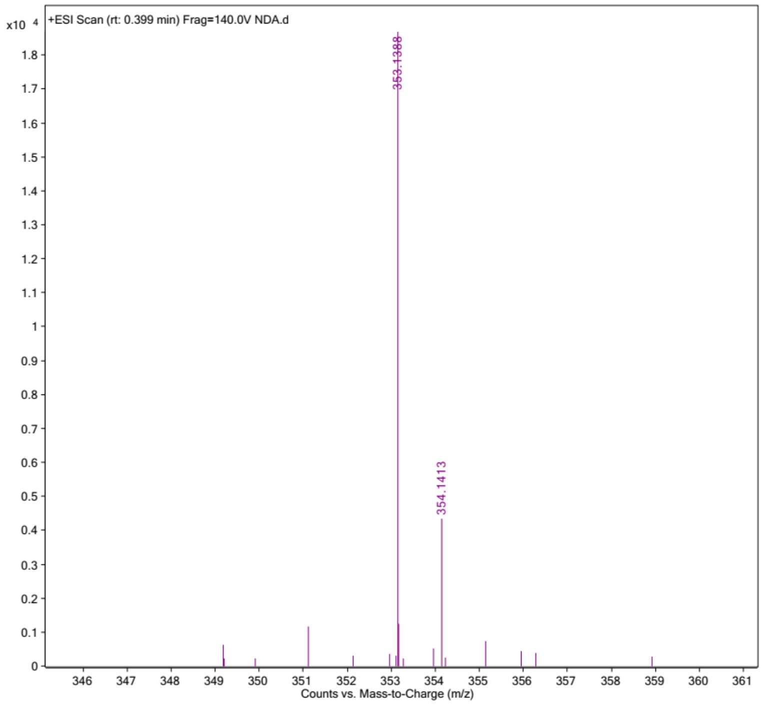

^{1}H-NMR

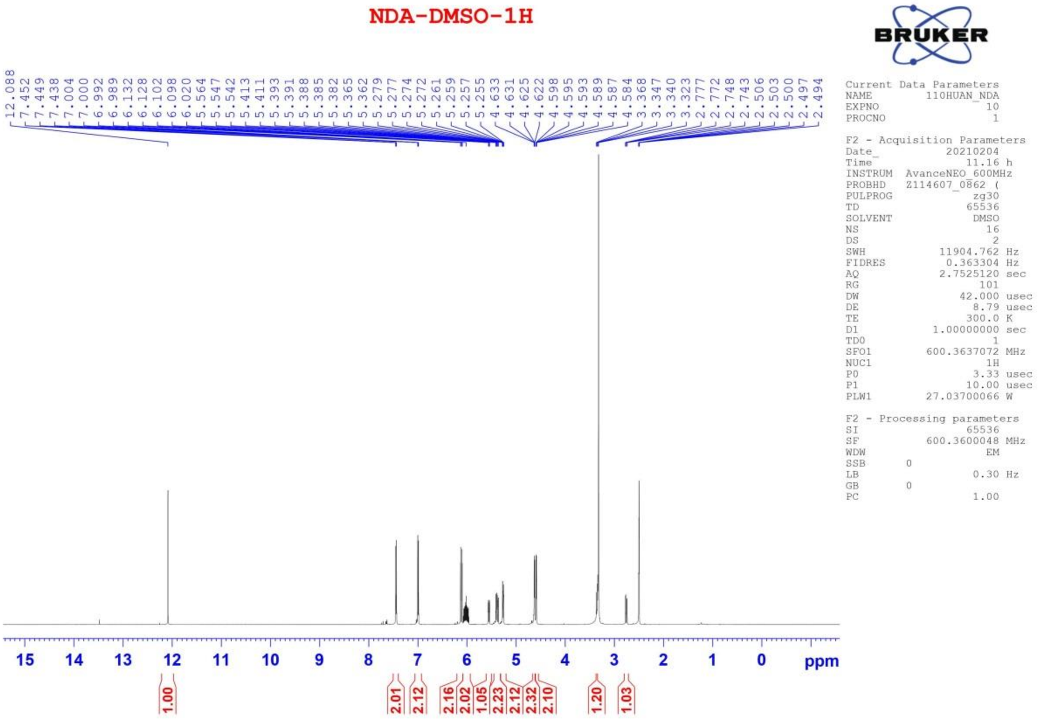
NDA-DMSO-1H
BRUKER
12.088
7.452
7.449
7.438
7.004
7.000
6.992
6.989
6.132
6.128
6.102
6.098
6.020
5.564
5.547
5.542
5.413
5.411
5.393
5.391
5.388
5.385
5.382
5.365
5.362
5.279
5.277
5.274
5.272
5.261
5.259
5.257
5.255
4.633
4.631
4.625
4.622
4.598
4.595
4.593
4.589
4.587
4.584
3.368
3.347
3.340
3.323
2.777
2.772
2.748
2.743
2.506
2.503
2.500
2.497
2.494
Current Data Parameters
NAME 110HUAN_NDA
EXPNO 10
PROCNO 1
F2 - Acquisition Parameters
Date_ 20210204
Time 11.16 h
INSTRUM AvanceNEO_600MHz
PROBHD Z114607_0862 (
PULPROG zg30
TD 65536
SOLVENT DMSO
NS 16
DS 2
SWH 11904.762 Hz
FIDRES 0.363304 Hz
AQ 2.7525120 sec
RG 101
DW 42.000 usec
DE 8.79 usec
TE 300.0 K
D1 1.00000000 sec
TD0 1
SFO1 600.3637072 MHz
NUC1 1H
P0 3.33 usec
P1 10.00 usec
PLW1 27.03700066 W
F2 - Processing parameters
SI 65536
SF 600.3600048 MHz
WDW EM
SSB 0
LB 0.30 Hz
GB 0
PC 1.00
15 14 13 12 11 10 9 8 7 6 5 4 3 2 1 0 ppm
1.00
2.01
2.12
2.16
2.02
1.05
2.23
2.12
2.32
2.10
1.20
1.03

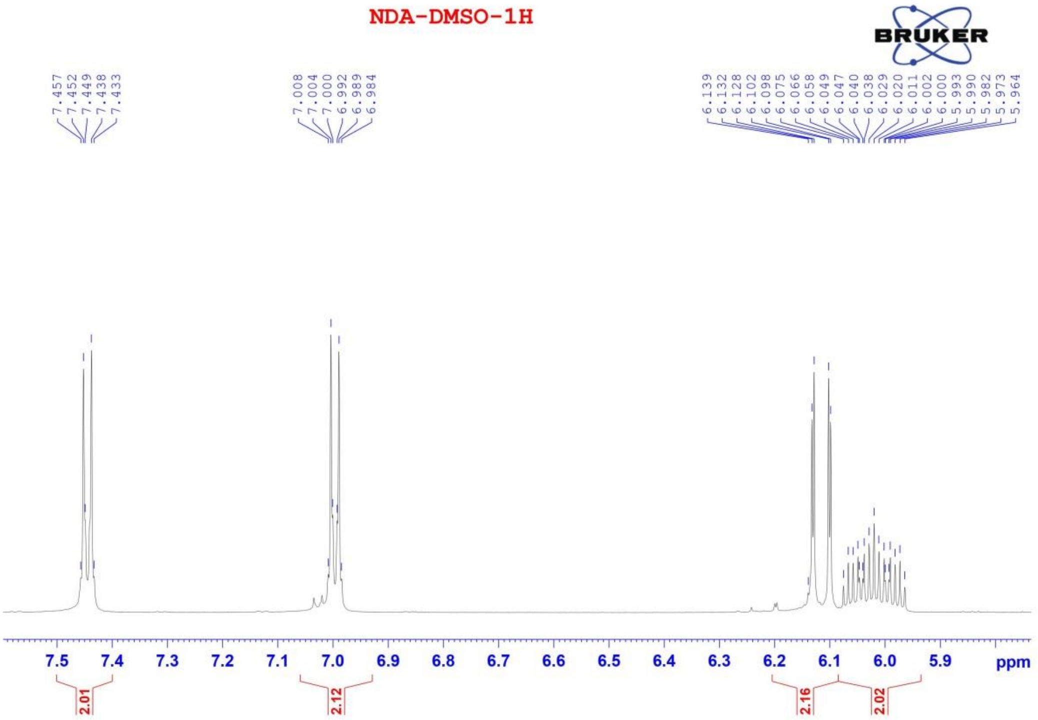
NDA-DMSO-1H
BRUKER
7.457
7.452
7.449
7.438
7.433
7.008
7.004
7.000
6.992
6.989
6.984
6.139
6.132
6.128
6.102
6.098
6.075
6.066
6.058
6.049
6.047
6.040
6.038
6.029
6.020
6.011
6.002
6.000
5.993
5.990
5.982
5.973
5.964
7.5 7.4 7.3 7.2 7.1 7.0 6.9 6.8 6.7 6.6 6.5 6.4 6.3 6.2 6.1 6.0 5.9 ppm
2.01
2.12
2.16
2.02

^{13}C-NMR

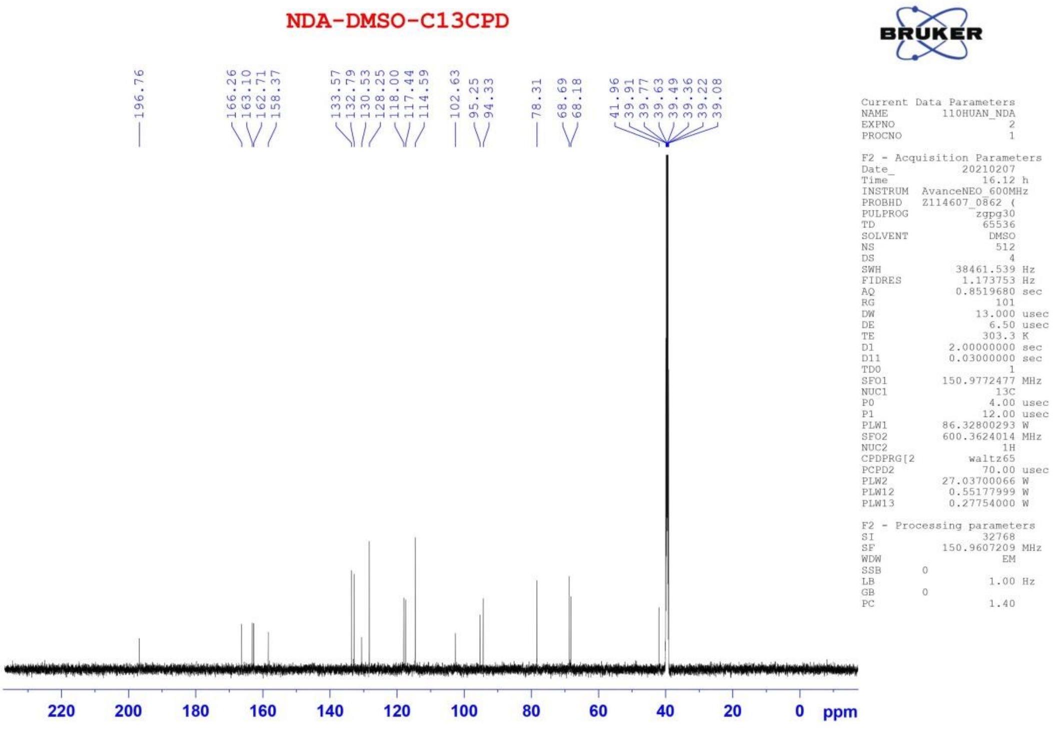
NDA-DMSO-C13CPD
BRUKER
196.76
166.26
163.10
162.71
158.37
133.57
132.79
130.53
128.25
118.00
117.44
114.59
102.63
95.25
94.33
78.31
68.69
68.18
41.96
39.91
39.77
39.63
39.49
39.36
39.22
39.08
Current Data Parameters
NAME 110HUAN_NDA
EXPNO 2
PROCNO 1
F2 - Acquisition Parameters
Date_ 20210207
Time 16.12 h
INSTRUM AvanceNEO_600MHz
PROBHD Z114607_0862 (
PULPROG zgpg30
TD 65536
SOLVENT DMSO
NS 512
DS 4
SWH 38461.539 Hz
FIDRES 1.173753 Hz
AQ 0.8519680 sec
RG 101
DW 13.000 usec
DE 6.50 usec
TE 303.3 K
D1 2.00000000 sec
D11 0.03000000 sec
TD0 1
SFO1 150.9772477 MHz
NUC1 13C
P0 4.00 usec
P1 12.00 usec
PLW1 86.32800293 W
SFO2 600.3624014 MHz
NUC2 1H
CPDPRG[2 waltz65
PCPD2 70.00 usec
PLW2 27.03700066 W
PLW12 0.55177999 W
PLW13 0.27754000 W
F2 - Processing parameters
SI 32768
SF 150.9607209 MHz
WDW EM
SSB 0
LB 1.00 Hz
GB 0
PC 1.40
220 200 180 160 140 120 100 80 60 40 20 0 ppm

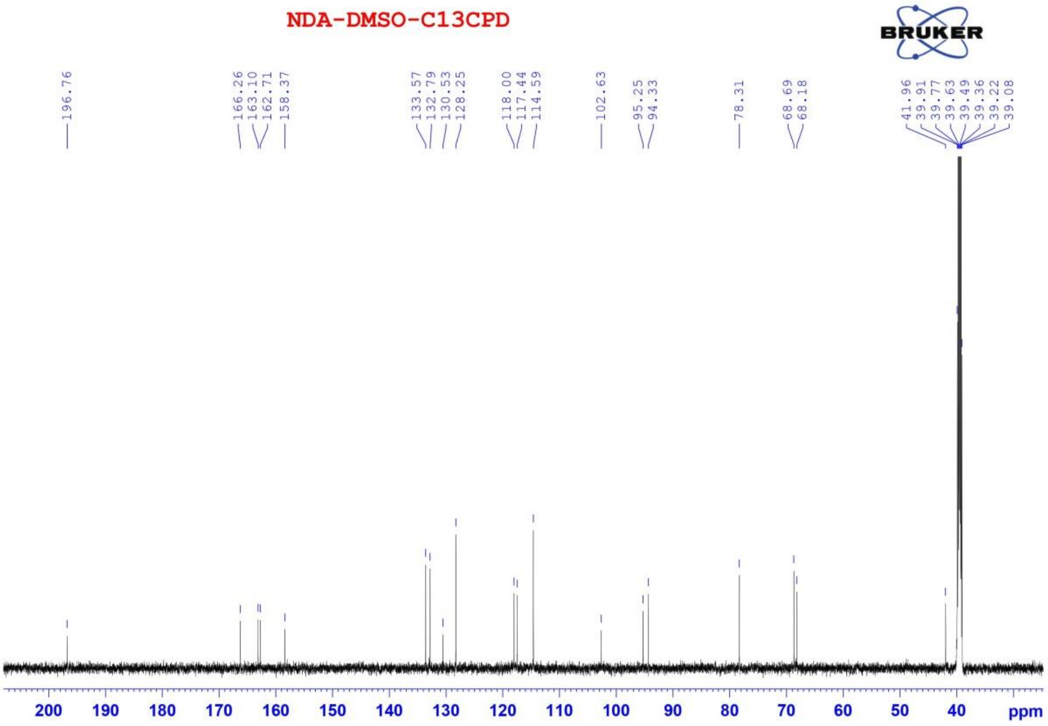
NDA-DMSO-C13CPD
BRUKER
196.76
166.26
163.10
162.71
158.37
133.57
132.79
130.53
128.25
118.00
117.44
114.59
102.63
95.25
94.33
78.31
68.69
68.18
41.96
39.91
39.77
39.63
39.49
39.36
39.22
39.08
200 190 180 170 160 150 140 130 120 110 100 90 80 70 60 50 40 ppm
