# Supplementary material for: Synthesis and Evaluation of the Acetylcholinesterase Inhibitory Activities of Some Flavonoids Derived from Naringenin
Source: ScientificWorldJournal. 2021 Nov 30;2021:4817900. doi: 10.1155/2021/4817900 (PMC8651387; doi:10.1155/2021/4817900)

Table S8. Spectral data of compound 8

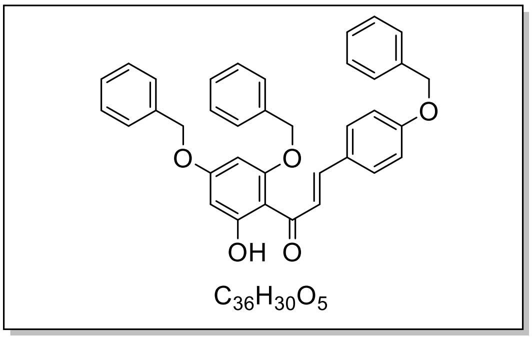

UV

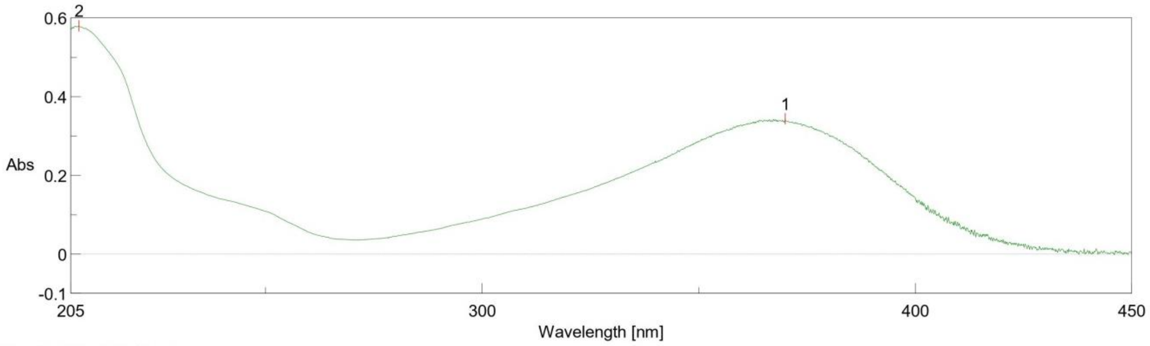

[Result of Peak Picking]

No.	Position	Intensity	No.	Position	Intensity	No.	Position	Intensity
1	370	0.343353	2	206.9	0.579749			

IR

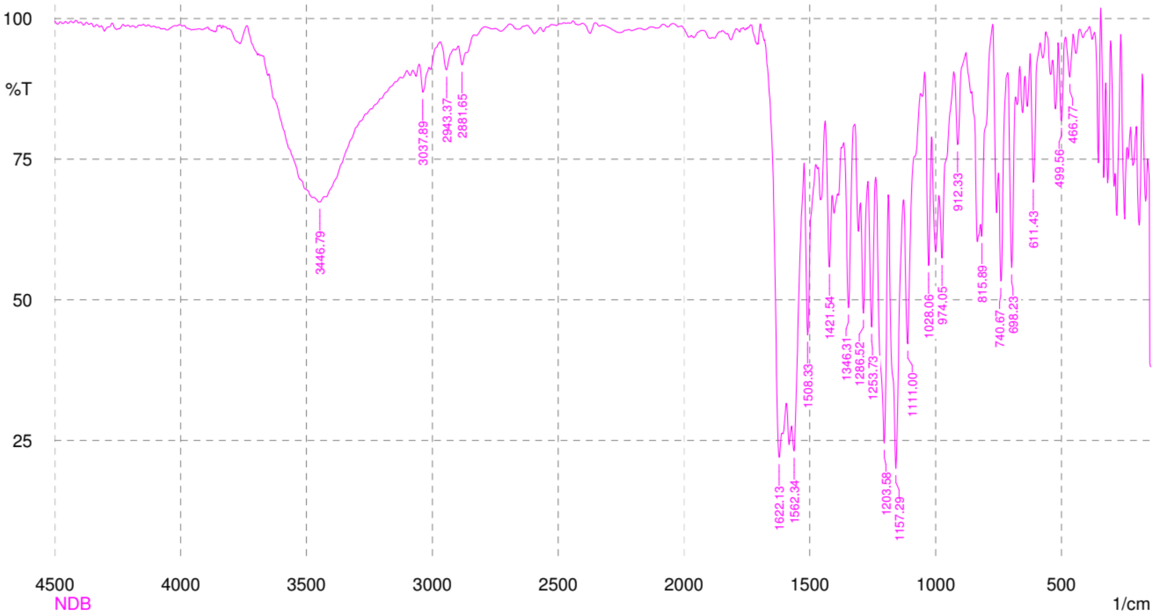

HR-MS

Qualitative Analysis Report

**Data File**	NDBc.d	**Sample Name**	NDBc
**Sample Type**	Sample	**Position**	P1-A8
**Instrument Name**	Instrument 1	**User Name**	
**Acq Method**	Cot ngan - MSMS_Pos.m	**Acquired Time**	01/07/2021 3:39:53 PM
**IRM Calibration Status**	Success	**DA Method**	COTNGAN.M.m
**Comment**			
**Sample Group**		**Info.**	
**Stream Name**	LC 1	**Acquisition SW Version**	6200 series TOF/6500 series Q-TOF B.06.01 (B6172 SP1)

User Chromatograms

**Fragmentor Voltage**	140	**Collision Energy**	0	**Ionization Mode**	ESI

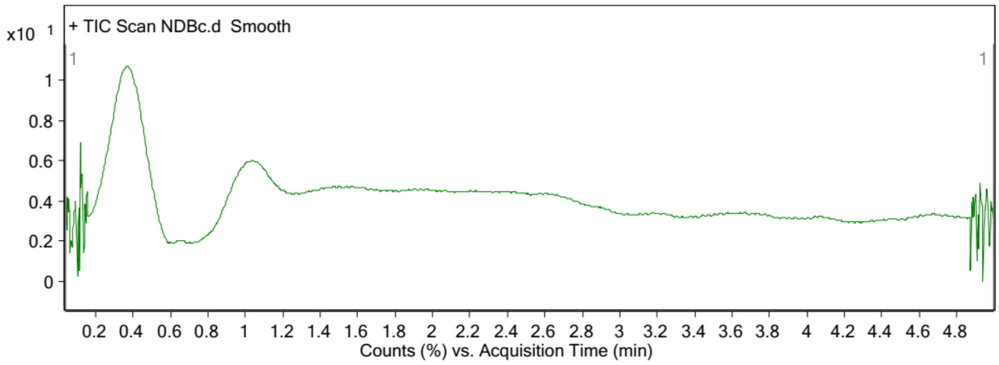

Qualitative Analysis Report

User Spectra

**Fragmentor Voltage**	**Collision Energy**	**Ionization Mode**
140	0	ESI

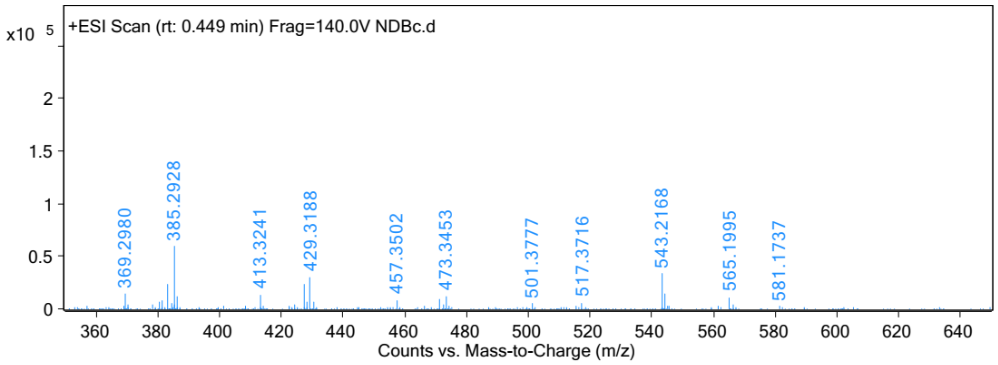

**Sample Name**	NDBc	**Position**	P1-A8	**Instrument Name**	Instrument 1
**User Name**		**Inj Vol**	2	**InjPosition**	
**Sample Type**	Sample	**IRM Calibration Status**	Success	**Data Filename**	NDBc.d
**ACQ Method**	Cot ngan - MSMS_Pos.m	**Comment**		**Acquired Time**	01/07/2021 3:39:53 PM

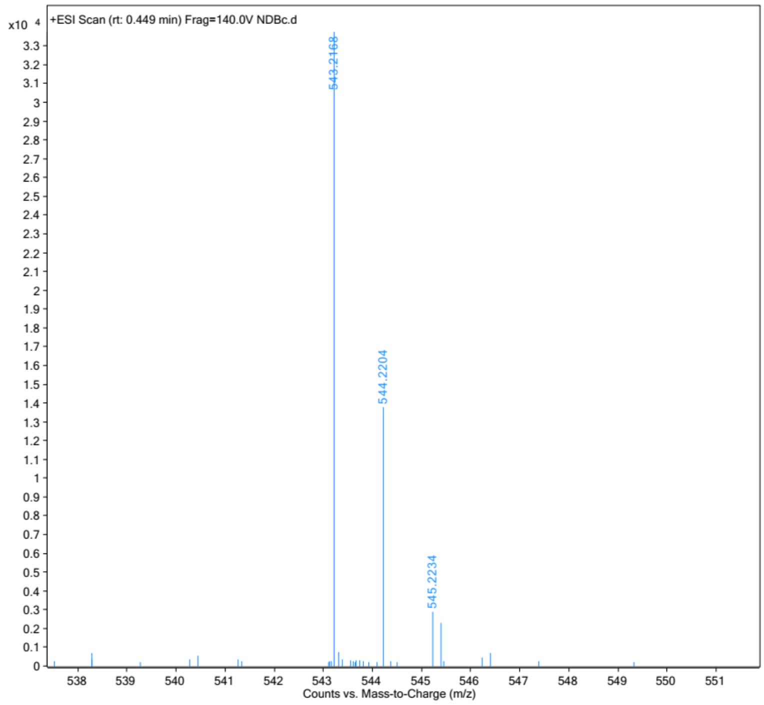

^{1}H-NMR

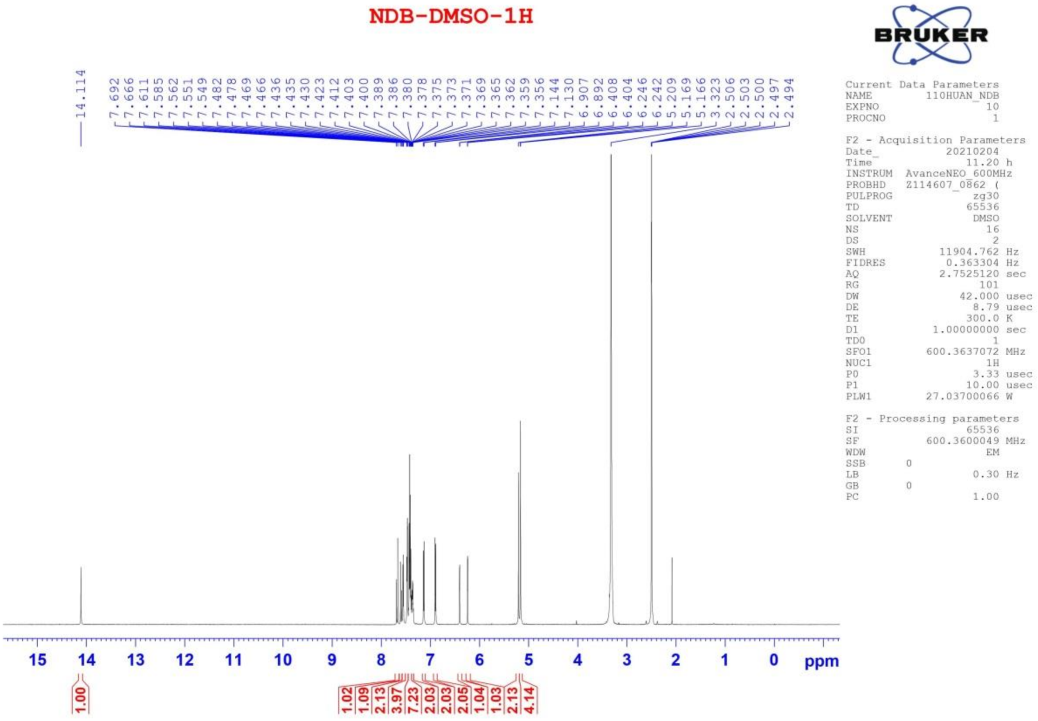

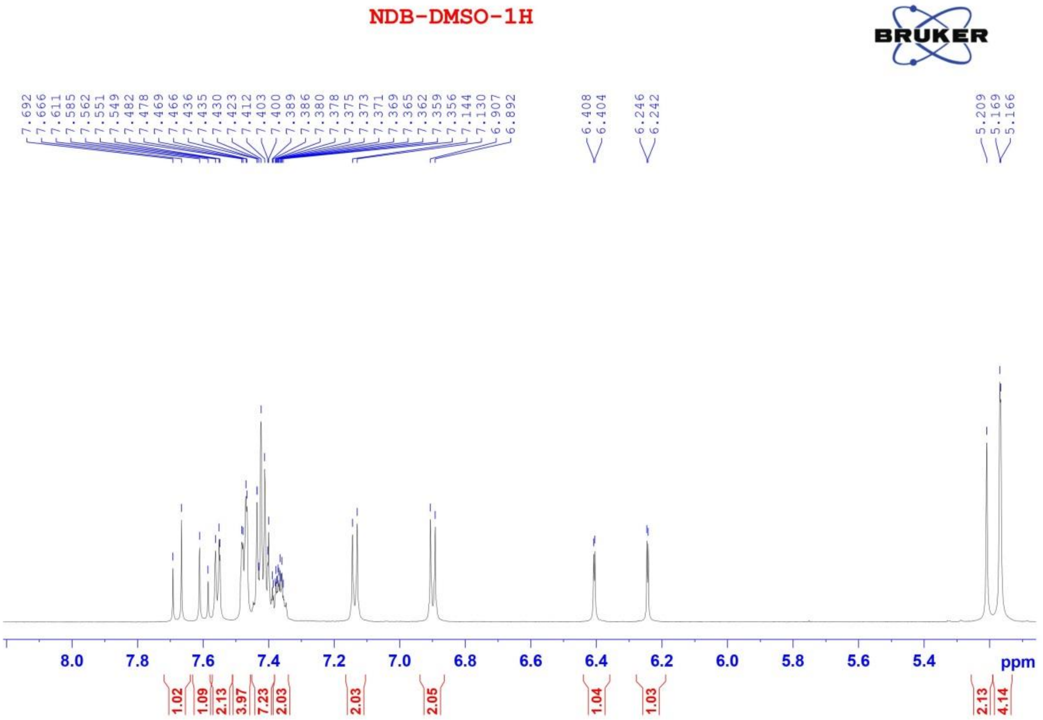

^{13}C-NMR

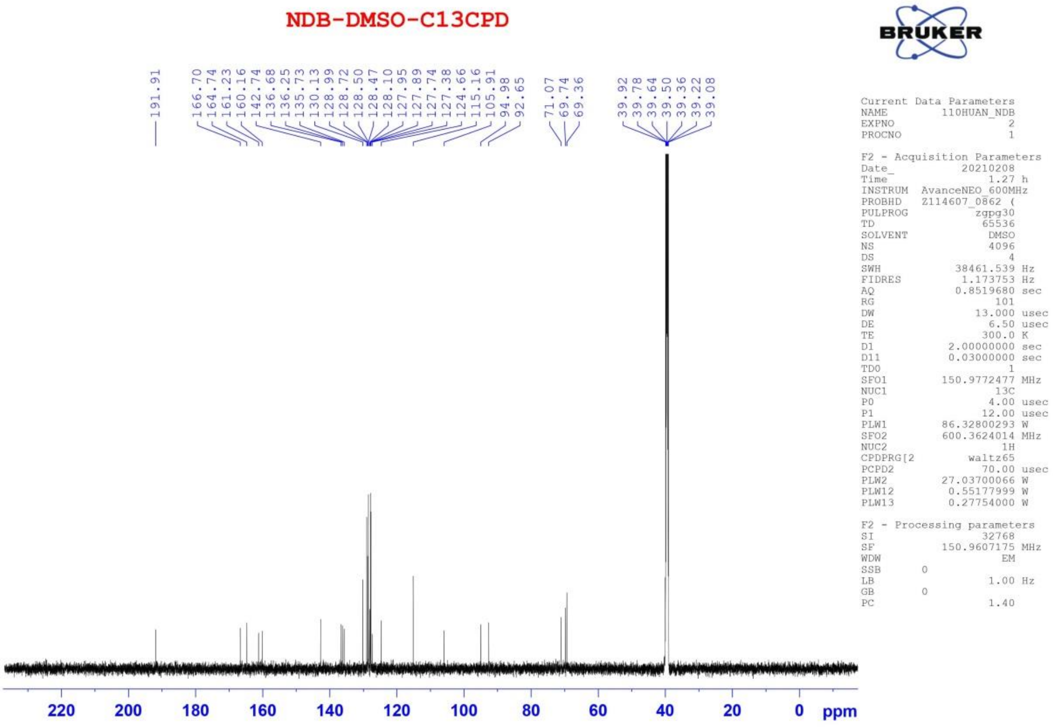

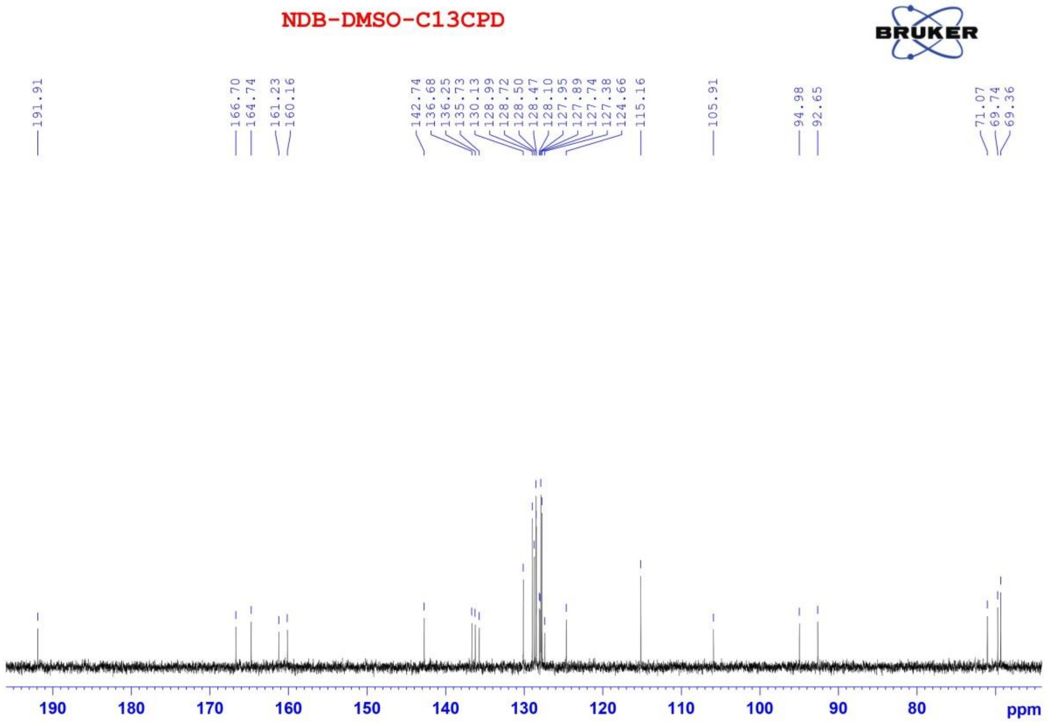

Supplement: Supplementary Materials — Tables S1–S9: spectral data of compounds 1–9. Figure S1: mode of ligand interaction of compound 2 with AChE. Figure S2: mode of ligand interaction of compound 4 with AChE. Figure S3: mode of ligand interaction of compound 5 with AChE. Figure S4: mode of ligand interaction of naringenin with AChE. Table S10: pharmacokinetics, drug-likeness, and medicinal chemistry evaluation of studied compounds by SwissADME online service. Table S11: predicted toxicities of studied compounds by ProTox-II web server. [file 4817900.f1.zip › 4817900.f1/Table S8. Spectral data of compound 8.pdf]
